# The Versatility of Biological Field-Effect Transistor-Based Biosensors (BioFETs) in Point-of-Care Diagnostics: Applications and Future Directions for Peritoneal Dialysis Monitoring

**DOI:** 10.3390/bios15030193

**Published:** 2025-03-18

**Authors:** Quan Wang, Zi-An Zhao, Ke-Yu Yao, Yuk-Lun Cheng, Dexter Siu-Hong Wong, Duo Wai-Chi Wong, James Chung-Wai Cheung

**Affiliations:** 1 Department of Biomedical Engineering, Faculty of Engineering, The Hong Kong Polytechnic University, Hong Kong; 2 Department of Medicine, Alice Ho Miu Ling Nethersole Hospital, Hong Kong; 3 School of Medicine and Pharmacy, Ocean University of China, Qingdao 266003, China; 4 Research Institute for Smart Ageing, The Hong Kong Polytechnic University, Hong Kong

**Keywords:** peritoneal dialysis, BioFETs, biomarkers, point-of-care, chronic kidney disease

## Abstract

Peritoneal dialysis (PD) is a vital treatment for end-stage renal disease patients, but its efficacy is often compromised by complications such as infections and peritoneal fibrosis. Biological field-effect transistors (BioFETs) present a promising solution for rapid, sensitive, and non-invasive detection of indicators and biomarkers associated with these complications, potentially enabling early intervention. However, BioFETs are yet to be adopted for PD monitoring. This review presents a forward-looking analysis of the capacity and potential integration of BioFETs into PD management systems, highlighting their capacity to monitor both routine indicators of dialysis efficiency and metabolic status, as well as specific biomarkers for complications such as inflammation and fibrosis. We examine the challenges in adapting BioFETs for PD applications, focusing on key areas for improvement, including sensitivity, specificity, stability, reusability, and clinical integration. Furthermore, we discuss various approaches to address these challenges, which are crucial for developing point-of-care (PoC) and multiplexed wearable devices. These advancements could facilitate continuous, precise, and user-friendly monitoring, potentially revolutionizing PD complication management and enhancing patient care.

## 1. Introduction

Peritoneal dialysis (PD) is a home-based therapy for kidney failure [1], which is primarily used for patients with chronic kidney disease (CKD) to help preserve the residual renal function, particularly in the late stages—end-stage renal disease (ESRD)—when kidney function drops to 10–15% of its normal capacity [2]. CKD affects approximately 700 million people worldwide [3], representing about 10% of the population [4]. PD accounts for around 11% of global dialysis therapies [5] and PD patients often report better health-related quality of life [6]. This is largely due to the greater autonomy and flexibility PD offers [7], allowing patients to perform the treatment at home, especially during the initial years of treatment [8].

Despite these advantages, PD is primarily challenged by infection and peritoneal fibrosis (PF). Infection-related problems, including peritonitis and catheter infection, occur in 20% to 40% of cases depending on the region [9]. Patients undergoing prolonged PD treatment are at risk of developing PF, with incidence rates ranging from 0.5% to 19.4% depending on the duration of therapy. As PF progresses, it leads to a decline in dialysis efficiency, and affected patients may no longer be able to use PD. These patients are then forced to return to hemodialysis (HD), which is more cumbersome and costly [10,11]. Additionally, patient-specific factors, including reduced peritoneal membrane longevity, declining ultrafiltration capacity [12], overhydration [13], and hypokalemia [6], further contribute to the eventual treatment failure. Therefore, real-time monitoring and early detection of complications are crucial for delivering targeted interventions and minimizing the risk of PD failure [14].

To meet these challenges, PD patients undergo regular prescriptions and follow-ups, including assessments of peritoneal transport status, solute clearance, and screening for complications [15]. Peritoneal biopsy requires surgical procedures to obtain peritoneal tissue, making it an invasive procedure [16,17]. The peritoneal equilibration test (PET) is commonly used to evaluate the peritoneal solute transfer rate (PSTR) and optimize solute clearance according to the kidney status [18]. However, this is a time-consuming process, as it involves instilling dialysate fluid into the peritoneal cavity and collecting samples at specific intervals over a 4 h period to monitor the transfer of solutes across the peritoneal membrane [19]. Non-invasive and efficient alternatives focus on the analysis of peritoneal dialysis effluent (PDE), which serves as a rich source of indicators and biomarkers, such as glucose, urea, creatinine [16], cancer antigen 125 (CA125) [17], and lnterleukin-6 (IL-6) [20], which can help predict clinical PD outcomes [21].

The International Society of Peritoneal Dialysis (ISPD) recommends screening PDE through cell culture, Gram stain, white blood cell count, and clinical feature observation [22]. However, these methods are time-consuming and complex to perform, leading to the use of empirical antibiotics as an initial treatment before the infection source is confirmed [23]. This delay poses a challenge for timely intervention, as the risk of complications and death increases by 5.5% for each additional hour of delay in treatment [23]. So researchers have been exploring proteomics and metabolomics approaches for PDE analysis [24], including nuclear magnetic resonance (NMR)-based identification of metabolites [25], as well as proteomics techniques such as Western blot [26], ELISA, and two-dimensional differentiation gel electrophoresis [10]. Other novel technologies, such as polymerase chain reaction/electrospray ionization mass spectrometry (PCR/ESI-MS) and 16S rRNA gene sequencing, have been developed to quickly detect pathogens in PDE as well [27]. However, PDE metabolomics may differ significantly depending on patient peritoneal type [25]. Proteomics are often interfered by high-abundance proteins, necessitating sample pretreatment, which slows clinical translation [10]. PCR/ESI-MS lacks accuracy [28], and gene sequencing struggles to differentiate pathogens with high genetic similarity [29]. Furthermore, all these techniques require sophisticated instruments and laboratory conditions, which makes them less accessible and incapable of being used for point-of-care (PoC) diagnostics for PD patients. This limitation highlights the need for efficient, non-invasive, and label-free detection platforms that can enable PoC testing, thereby minimizing the risk of death or complications caused by delays in infection treatment [30].

In response to the limitations of current diagnostic methods, transistor-based biosensors emerged as a promising avenue for advancing PD monitoring. These transistors primarily fall into two categories: bipolar junction transistors (BJTs) and biological field-effect transistors (BioFETs). BioFETs consist of a semiconductor channel connecting source and drain electrodes, with a gate electrode that modulates the channel’s conductivity via an applied voltage [31]. BJTs, on the other hand, are three-terminal devices composed of two p-n junctions, where a small current injected into the base controls a larger current between the collector and emitter. While BioFETs rely on voltage-controlled modulation of channel conductivity, BJTs offer current-controlled amplification [32]. Recent research suggests that BJTs can surpass BioFETs in certain performance aspects, including achieving simpler calibration curves independent of applied voltage, lower signal-to-noise ratios, and higher sensitivity. For example, researchers demonstrated a heterojunction MoTe_2_/GeSe/MoTe_2_ BJT capable of highly sensitive detection of diverse proteins and DNA, achieving a limit of detection (LOD) of 5 pM for streptavidin within 10 s—a performance exceeding many existing FET-based detection methods [33]. Similarly, BJTs have been used to detect C-reactive protein (CRP) with ultra-low detection limits (1 pmol/L), surpassing the sensitivity of many FET-based CRP sensors [34,35]. Despite these advantages, the application of BJTs in the medical field, and particularly in biosensing, remains less prevalent than that of FETs, with current BJT applications primarily focused on areas such as X-ray radiation therapy [36], pressure sensing [37], and protein detection [33]. Although BJTs offer potential for point-of-care (PoC) diagnostics and demonstrate superior performance in some areas, including the possibility of atomic-layer thickness [33], a critical challenge lies in scaling down BJT fabrication to below 100 nanometers in an easy method for mass production and widespread commercialization [38]. Given that achieving ultra-low detection limits often depends on maximizing sensor surface area, highly miniaturized BioFETs may offer a more practical approach in this specific context. Therefore, this review centers on BioFETs, exploring their potential for addressing the unique challenges of PoC PD monitoring while acknowledging the exciting possibilities offered by BJT technology. While PET is valuable for assessing long-term peritoneal membrane function, BioFETs could provide real-time monitoring of key biomarkers, alerting clinicians to acute changes that might be missed by infrequent PET testing. BioFETs could offer a bacterial detection limit over 10,000 times lower than electrochemical impedance spectroscopy (EIS) and matrix-assisted laser desorption ionization time-of-flight mass spectrometry (MALDI-ToF) using the same modified surfaces [39]. For PD patients, BioFETs-based monitoring is non-invasive, while the lower manufacturing costs make them an affordable option [40]. The miniaturization capabilities of BioFETs enable PoC applications [41], allowing patients to perform home-based self-testing and see the results directly from wireless communication modules such as an app [42,43]. These advantages support personalized treatment plans, as regular monitoring of residual renal function can be tailored to meet the unique needs of individuals [44].

BioFETs have been successfully applied in various fields [45], demonstrating their effective capabilities for integration with existing PD monitoring. These applications mainly include antigen detection for communicable diseases [46], particularly COVID-19 [47] and influenza [48], as well as biomarker detection for non-communicable diseases [49], mainly cancers [50], cardiovascular conditions [51,52], and neurodegenerative disorders [53]. Additionally, BioFETs are utilized in drug screening through monitoring cell membrane potential changes [54]. Despite their success in other areas, their direct use in PD monitoring remains limited and underexplored [55,56]. Since PD monitoring requires long-term follow-up [20], the integration of BioFETs faces several challenges, including the stringent demands on stability, sensitivity, specificity, and the need for consistent performance. Many BioFETs remain at the laboratory stage [57], and the complex composition of PDE effluent and bodily fluid [58], along with issues related to long-term device performance [59], manufacturing costs [31], and system integration [60], further complicates their application in routine PD monitoring. Several reviews explored various aspects of BioFETs, including their configuration [61], transducing materials, probes [62], and applications in wearable healthcare technologies [52]. However, the potential of BioFETs specifically for PD monitoring remains largely unexplored.

**Figure 1:**
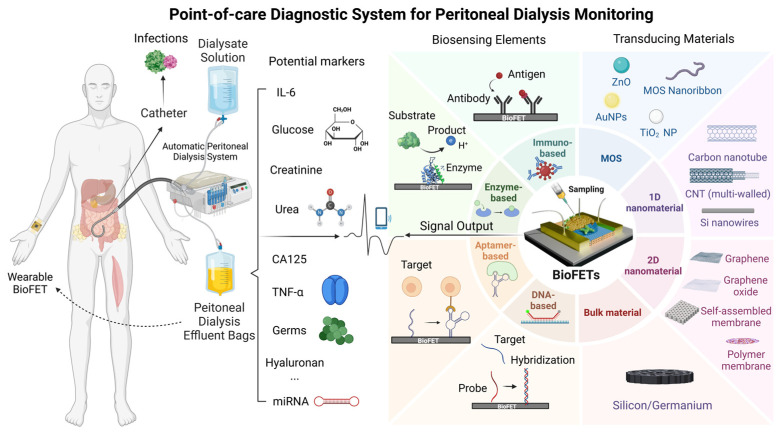
Schematic overview of BioFETs components and their applications in PD monitoring.

The primary objective of this review is to critically evaluate the adaptability of BioFETs for PD monitoring, focusing on three key areas: (1) the design and optimization of BioFETs components (probes, transducing materials) for PD-specific biomarker detection; (2) the technical and clinical barriers to integrating BioFETs into PD workflows; and (3) emerging strategies (e.g., multiplexing, PoC) to address these challenges. By synthesizing advances in BioFETs engineering with unmet clinical demands in PD, this work delineates a roadmap for translating laboratory innovations into practical, patient-centric diagnostic tools. The successful integration of BioFETs into PD management could revolutionize personalized medicine, offering tailored interventions for individual patients and greatly improving the quality of life and long-term outcomes for those with ESRD undergoing PD. To provide a conceptual overview, Figure 1 illustrates the interplay between the core components of BioFETs and their applications in PD monitoring.

The outline of this review is as follows: The next section delves into the core components of BioFETs, focusing on biosensing elements and transducing materials, which are crucial for improving device performance. Section 3 and Section 4 explore the functions of BioFETs applicable to PD monitoring and assess the technical and clinical barriers hindering their integration into PD management. Lastly, this work explores solutions to overcome these challenges for the improvement of usability and clinical applicability, including optimized device design, multiplexing capabilities, wearable BioFETs for portable PoC testing, and advances in fabrication techniques. These innovations demonstrate how BioFETs can be effectively integrated with other approaches to enhance PD monitoring.

## 2. Overview of BioFETs

### 2.1. Introduction of BioFETs

**Figure 2:**
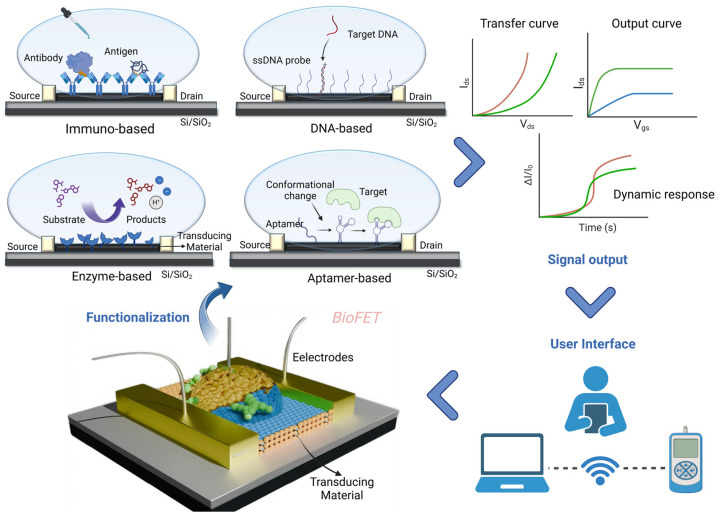
Schematic of BioFETs structure and workflow.

BioFETs are fundamentally voltage-controlled amplifiers [63] widely employed in biosensor design for analyte detection, with applications spanning environmental monitoring, agriculture, the food industry, and particularly the medical field [61]. In typical BioFETs, the source and drain electrodes are connected via semiconductor channels, with a gate electrode controlling the flow of charge carriers by applying a bias potential [64]. Biosensing elements are immobilized on the semiconductor channels of BioFETs to specifically capture the target molecules [62]. When a target binds to a probe, this interaction induces a change in the charge carrier density within the channel, altering its conductivity. This shift in conductivity, driven by the binding reaction, can be easily detected and measured, allowing for the sensitive detection of specific biomolecular interactions [49,61,65]. Figure 2 illustrates the operational workflow of BioFETs, encompassing their structure, functionalization, signal output, and user interface.

BioFETs enable label-free, real-time electrical detection of biomolecules, offering advantages in direct signal transduction without requiring fluorescent labeling or optical instrumentation [66]. While other systems, such as fluorescence-based systems, also excel in single-molecule sensitivity, BioFETs are particularly suited for applications demanding miniaturization, continuous monitoring, and integration with portable electronic systems [31,42,67]. Taking these advantages, BioFETs have already been applied in various healthcare areas, including precision medicine and the early detection of biomarkers such as urea [55], glucose [68], and creatinine [69]. However, the commercial deployment of BioFETs remains limited due to issues such as the development of disposable devices, difficulties in large-scale production [57], low biocompatibility [70], and especially Debye screening effect [71]. BioFETs rely on detecting the electrostatic potential of charged biomolecules, but in physiological solutions, mobile ions rapidly screen these charges over a characteristic distance called the Debye length (κ^−1^). If the target analyte is located beyond this length—typically <1 nm in physiological conditions—its signal is effectively shielded, limiting the sensor’s ability to detect larger biomolecules and reducing sensitivity in clinical applications [72,73]. So, physiological samples analyzed using BioFETs usually require dilution or pretreating, but recent advancements proposed solutions to streamline this, as is later detailed in the following sections.

**Figure 3:**
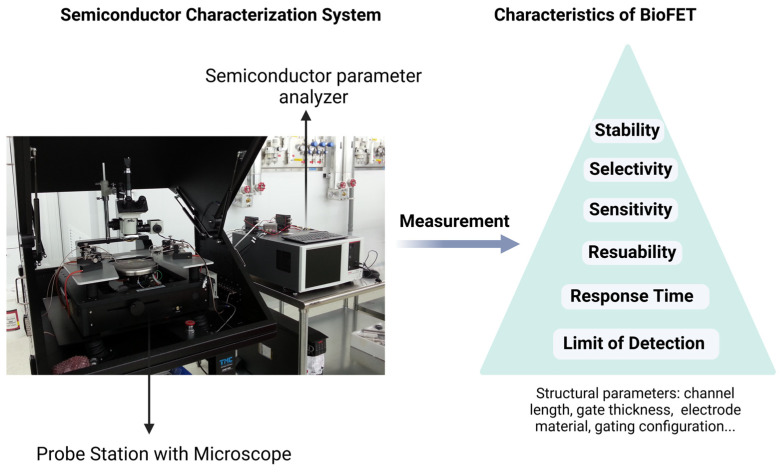
Evaluation of the performance of BioFETs and the characteristics of BioFETs through a semiconductor characterization system.

The performance of BioFETs is also determined by several other factors. Depending on the biometric event and sensor operation parameters, BioFETs sensitivity formulation can be expressed as [74]:

(1) $$\frac{dI_{D}}{I_{D}}=dC\frac{d\sigma }{dC}\cdot \frac{dV_{EG}}{d\sigma }\cdot \frac{dI_{D}/dV_{EG}}{I_{D}}$$

where *dC* is the change in the analyte concentration, *dσ* is the change in the sensor conductivity due to the charge transfer process, and *dV_EG_* refers to gate voltage change, which results in the variation in drain current I_D_. The first half of the formula represents a change in the sensor conductivity caused by a biological reaction, expressing the response to the presence of the analyte. The second half represents a change in conductivity during signal transduction that changes the gate threshold voltage, resulting in a detectable change in leakage current I_D_. Thus, biosensing elements and transducing materials can be spotted as two main factors when designing BioFETs [62], which determine their sensitivity, selectivity, limit of detection (LOD), stability, and reusability [75]. Structural parameters including channel length, gate insulator thickness, electrode metal type, channel material thickness, and gating structure contribute to the device performance as well (Figure 3) [76]. While general designs of BioFETs are covered in other literature [74], our emphasis here is on the biosensing elements and transducing materials employed in BioFETs for the PD-related biomarkers detection, demonstrating their suitability for PD diagnostics.

### 2.2. Biosensing Elements

**Figure 4:**
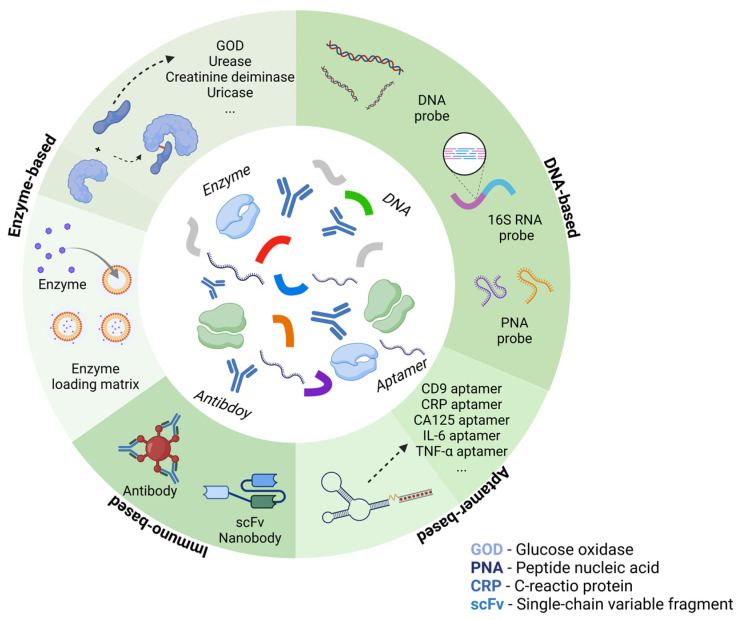
Common bioreceptors used in BioFETs for PD-related biomarkers monitoring.

**Figure 5:**
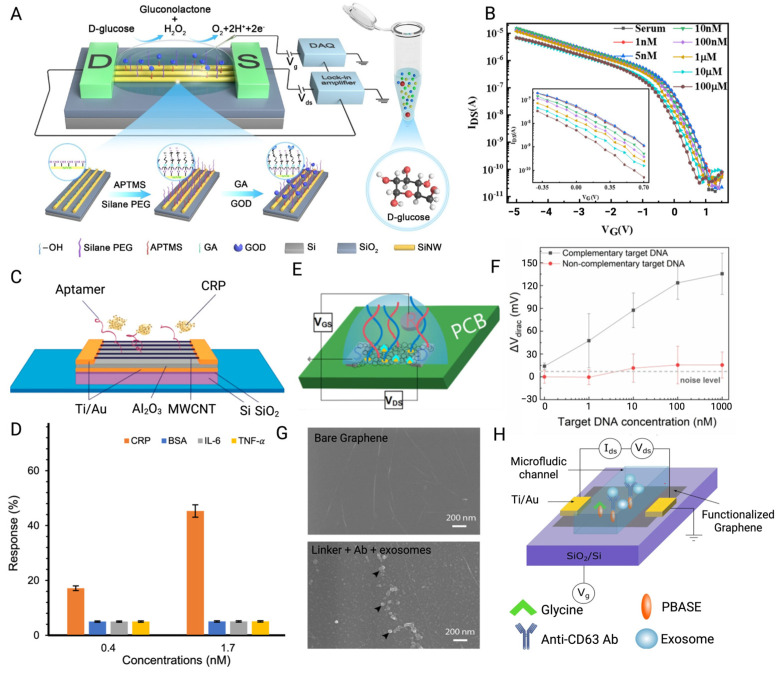
(**A**) Glucose oxidase and PEG layers were co-modified on the SiNW surface for the glucose detection through catalytic principle. (**B**) Transfer curves of SiNW-BioFETs for detecting glucose in serum samples [77]. (**C**) Schematic of the MWCNT-FET modified with aptamer for capturing target CRP. (**D**) The response demonstrated by BioFETs indicated its high affinity for the target CRP despite interference from three other proteins commonly present during PD inflammation [78]. (**E**) Schematic of a PNA-based EGFET fabricated on PCB for DNA hybridization. Blue lines are the PNA probes, while the red ones are the target DNA. (**F**) Mean values of *V_dirac_* shifts of BioFETs used for DNA hybridization (bars represent standard deviations of 5 BioFETs) [79]. (**G**) SEM images of bare graphene and graphene layer conjugated with probes and exosomes. (**H**) Schematic of a microfluidic GFET modified with CD63 antibody for detecting the surface protein of exosome [80] (images used under Creative Commons Licenses).

BioFETs are highly sensitive to the surrounding environment but have low selectivity. To address this, biosensing elements which have high specificity for the target are usually immobilized on the BioFET surface through appropriate connectors to capture the targets [70]. Based on different recognition elements and binding principles, they can be divided into enzyme-based, DNA-based, aptamer-based, and immune-based FET biosensors (Figure 4). They have been used for the biomarker detection according to different mechanisms, for instance, enzyme-based BioFETs are used to detect glucose and urea through enzyme-catalyzed reactions; DNA probe-based BioFETs can identify pathogen genes during PD peritonitis through base complementarity; aptamer-based BioFETs can overcome the Debye screening length in PDE with a smaller size; immune reaction-based BioFETs can recognize cytokines or bacteria through specific antigen–antibody binding. This section selects the four most common probe types as the basis to illustrate how they can be applied for PD monitoring (Figure 5).

#### 2.2.1. Enzyme-Based BioFETs

Enzyme-based BioFETs (EnFETs) have been broadly adapted in the detection of PD-related biomarkers, especially glucose [81,82]. EnFETs typically operate based on enzyme-catalyzed reactions, where enzymes immobilized on the channel surface facilitate the conversion of substrates into products. This reaction occurs within the enzyme membrane, inducing alterations in the accumulation of charged carriers at the gate surface, along with the generation or consumption of protons in the channel, leading to pH changes. These variations can then be used to quantify the target analyte [43]. In glucose detection, glucose oxidase (GOD) fixed on the EnFETs catalyzes the oxidation of glucose, generating positive charges. The accumulation of these positive charges on the gate of the n-type FET enhances the carrier concentration in the channel, leading to an increase in drain current, which enables the detection of glucose molecules [81]. Real-time urea detection in dialysis follows a similar principle. Urease was immobilized on a graphene-based EnFET using the EDC/NHA method to specifically recognize urea. In this system, urease catalyzes the hydrolysis of urea into carbon dioxide and ammonia, increasing the local pH and triggering a potential change within minutes [55]. This rapid response has shown significant potential for improving the efficiency of indirect urea clearance estimation for clinical PD monitoring.

These enzymes are popular because of their high specificity, availability [81], and even reversible reactions, which facilitate the reusability of BioFETs [83]. However, they are prone to deactivation when exposed to fluctuations in pH, temperature, and humidity [84]. This situation makes the immobilization of enzymes on BioFETs quite challenging, especially in biofluids such as PDE [85]. While the immobilization method and uniformity of immobilization can have a huge effect on the sensitivity fluctuations [81], new strategies have been conducted to deal with these problems. For examples, nanocomposites such as potassium-doped carbon nanotubes (CNT) have been used as the enzyme loading matrix [86], while Au nanoparticles (AuNPs) and polyelectrolytes have been employed for multilayer enzyme encapsulation to enhance system stability [87].

To improve the biocompatibility of the system, biomimetic materials are utilized as substitutes for traditional coatings. Silk fibroin (SF) hydrogel had been integrated with graphene BioFETs (GFETs) for glucose detection in physiological solution [84]. The enzymatically cross-linked SF serves as a carrier for GOD, effectively encapsulating the GFET while prolonging the lifetime of GOD and reducing non-specific adhesion of interferents. This device demonstrates high application potential with a LOD as low as 200 nM and a broad detection range from 1µM to 10 mM for glucose sensing, thereby fully encompassing the clinical diagnostic range for glucose [84]. Using a same-packaging approach, GOD was incorporated into an APTMS: PEG complex to create a porous biopolymer layer on the surface of silicon nanowire (SiNW) BioFETs. This “hybrid catalytic layer” maintains a sufficient density and activity of GOD on the BioFET surface while also increasing the Debye screening length, facilitating direct glucose detection in high-ionic-strength solutions. The device exhibits a dynamic glucose detection range of 10 nM~10 mM and a response time of less than 8 s, indicating high practical value, particularly in solutions with elevated ionic concentrations [77].

#### 2.2.2. DNA-Based BioFETs

In typical DNA probe-based BioFETs, single-stranded DNA (ssDNA) probes are usually immobilized on the BioFETs channel to hybridize the target DNA through complementary base pairing to alter the electron mobility and generate an electricity signal [70]. This approach overcomes the limitations of an optical-based real-time polymerase chain reaction (RT-PCR), which often requires toxic fluorescent dyes and unstable light source signals [51]. More importantly, it offers the potential for direct and label-free detection of DNA/RNA molecules without the need for time-consuming amplification processes [88,89], even down to the level of individual viral long RNA [90].

These properties make DNA-based BioFETs valuable for PD monitoring, including the detection of DNA or 16S rRNA for screening pathogens associated with prolonged catheter placement [91], mitochondrial DNA detection as indicators of peritoneal injury [92], and the assessment of elevated levels of pathological microRNA (miRNA) [93]. Notably, DNA-based BioFETs have been designed to detect Gram-negative bacterial (GNB) *E. coli* O157:H7, which is known to cause peritonitis. In this case, an ssDNA probe based on the *E. coli* intimin gene was covalently bound to the gold gate surface through a reduction reaction, enabling specific binding to the complementary DNA sequence (cDNA) in the target bacteria. The binding of negatively charged complementary DNA leads to the redistribution of the double-layer capacitance in the n-type BioFETs, resulting in a decrease in gate voltage and drain current, which enables the detection of the target [94].

Furthermore, studies have shown that it is possible to distinguish sequences that differ by a single base, which facilitates the identification of bacterial variants induced by peritoneal dialysis fluid (PDF), which are often underestimated [95]. In this context, ultra-thin film indium oxide has been utilized as the semiconductor channel material, with thiolated ssDNA probe fixed on the indium oxide channel via an amine–thiol linker to selectively capture the target sequence. The detection capabilities of BioFETs were then assessed by exposing them to non-complementary, fully complementary, and mismatched sequences, respectively. The results demonstrate that hybridization with fully complementary sequences led to a significant increase in the stable conductivity of the BioFETs, while the exposure to non-complementary and mismatched sequences resulted in minimal changes in conductivity following stabilization [96]. This differential signal response effectively enables the distinction among these three types of sequences and may facilitate the identification of pathogen variants during PD infection.

Though ssDNA probe has a high affinity with the cDNA, its large size usually increases the distance between the sensing surface and the target–probe reaction [97], which may lower the sensitivity due to the charge screening effect. Shorter probes, such as peptide nucleic acid (PNA), are introduced to replace ssDNA while it offers higher sensitivity. They are also named as peptide-based BioFETs to help the probe and target remaining within the Debye screening length [70], which should be approximately 0.7 nm to 2.2 nm in typical physiological samples [98]. The sensing performance of BioFETs modified with PNA and DNA probes, respectively, has been investigated to verify the enhanced sensitivity brought by smaller probe size. Additionally, the GFET modified with PNA probes demonstrated lower background noise and relatively higher selectivity than the latter. Its dynamic range and LOD were three orders of magnitude higher than those of the DNA-based GFET [99]. To integrate the benefits of this technology and lab-on-chip to develop PoC devices, it was further combined with the commercially manufactured printed circuit board (PCB) techniques. PCB electrodes can be directly used as the source, drain, and gate of FET to exempt the extra deposition process. Then, graphene ink can be drop-casted to form the FET channel, and PNA probes can then be fixed on it to recognize the target cDNA. This device has been applied for the label-free DNA detection of cell-free nucleic acid biomarkers and exhibited nearly twice the sensitivity compared with EGFET based on traditional silicon substrates [79].

#### 2.2.3. Aptamer-Based BioFETs

Aptamers, artificially synthesized ssDNA or RNA, can be chemically modified in a sequence-specific manner to improve their binding affinity to targets [100]. Notably, they can combine certain toxic substances that antibodies cannot detect [101]. In comparation to DNA probes, aptamers offer several advantages, including lower cost, higher stability, reduced susceptibility to interference from charged particles, and a smaller size [78,102]. These characteristics allow aptamers to retain their binding affinity even in high-ion concentration matrices [103], such as PDE. Within BioFETs, when an aptamer binds to its target, it typically transitions from a less structured two-dimensional (2D) form into a more organized and complex three-dimensional (3D) structure. This conformational change brings the target closer to the channel surface, improving the system conductivity [78].

For PD-related applications, aptamer-based BioFETs can be utilized to detect various biomarkers to understand the patient inflammation status; for instance, exosome surface transmembrane proteins differentiation 9 (CD9) protein, which reflects the dialysis efficiency [104], or C-reactive protein (CRP), which may indicate the membrane characteristics [105]. In the case of CRP detection, aptamers were immobilized on multi-wall carbon nanotube (MWCNT) BioFETs for rapid sensing. The BioFETs were easily functionalized by soaking overnight in an aptamer, where the aptamers initially adopted a stable 2D structure [78]. Upon the introduction of CRP to the sensing area, the negatively charged CRP induced positive charges in the MWCNT, increasing the number of free electron carriers and leading to a drain current change. Despite interference from PD-associated biomarkers such as IL-6 and TNF-α, the device rapidly detected changes in CRP concentration within minutes. It achieved a LOD as low as 0.017 mg/L, with a detection range that covers the CRP levels in the human body [78].

However, for practical applications, aptamers often require further optimization to enhance their binding affinity and stability, as they can be affected by factors such as ionic strength and interference [106]. To address this, additional selection processes, such as the systematic evaluation by exponential enrichment technique (SELEX) [107], are typically employed. In studies targeting the nucleic acid sequence of the CD9 protein, SELEX identified three sequences with high reproducibility and low ΔG values (Gibbs free energy) as the most effective candidates. Among these, CD9-26, which had the lowest dissociation constant (*K_d_*) and thus the highest affinity for the target, was selected as the optimal tool for capturing the target sequence. It was then covalently immobilized on MXene (Ti_3_C_2_T_x_) via the EDC/NHS method. MXene, with its numerous functional groups including hydroxyl and carboxyl groups, played a crucial role in facilitating the attachment and enhancing the overall performance of the sensing platform. The BioFETs exhibited a LOD of 6.41 × 10^2^ exosomes/mL and a detection range of 1 × 10^3^ to 1 × 10^7^ exosomes/mL in human serum, which significantly outperformed antibody-based methods [101]. Additionally, in comparation to traditional qRT-PCR [108], the proposed platform eliminates the need for exosome purification, separation, and gene extraction, offering a more economical and rapid alternative for clinical exosome detection.

#### 2.2.4. Immuno-Based BioFETs

In immune BioFETs, antibodies are usually used as probes and immobilized on FET channels. Changes in drain leakage current after antigen–antibody binding will generate detectable electrical signals since they are both charged molecules [70]. For PD-related applications, immune BioFETs demonstrated potential in bacterial screening [109] and biomarkers detection [80] as well. A BioFET-based sensing platform was proposed for detecting exosome membrane protein CD63 by integrating CD63 antibodies. The interaction between the antibodies and negatively charged exosomes induced the accumulation of positive charges in the graphene channel, leading to a shift in the Fermi level [80]. The method bypasses traditional exosome extraction and analysis techniques such as PCR and Western blot, avoiding the associated complexity and time consumption [110]. In experiments conducted by Kwong Hong Tsang et al. [80], this device achieved a LOD as low as 0.1 μg/mL. Albumin, another important PD biomarker, is closely linked to PD peritonitis and patient mortality [111]. Based on the same antigen–antibody reaction principle, albumin antibodies were immobilized on BioFETs for albumin detection [112]. In this study, the thickness of the transducing material was precisely controlled by using spray gun technology to obtain a single-layer film, which improved system sensitivity. This approach increased the surface area of transducing material and enhanced covalent binding during the biorecognition process [112].

**Figure 6:**
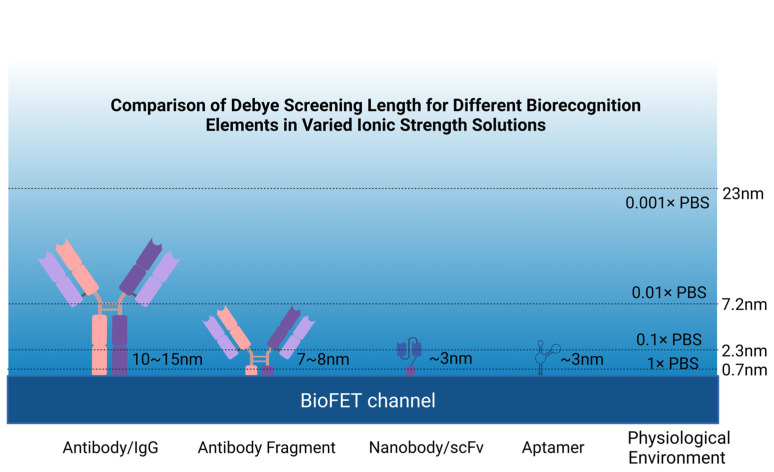
Comparison of various probes in solutions with different ionic strengths.

However, the size of antibody is generally larger than the Debye length, which results in reduced sensitivity [43], so many studies redesigned antibodies by retaining antibody-specific fragments. Single-chain variable fragment antibodies (scFv) have been used in the design of GFETs for the detection of pathogen antigens. They are obtained through the connection of the variable heavy chain and variable light chain domains of the parent IgG through short peptides, which have a smaller size and higher binding density. The result of binding experiments showed that the use of this probe increased the LOD by nearly 4000 times compared to traditional IgG GFETs [113]. Nanobodies have been incorporated into BioFETs as well. As single-domain antibodies that retain the specificity of the homologous antigen [114], they are usually less than 3 nm, much smaller than antibodies (~150 kDa, 15 nm), antibody fragments (Fab) (~50 kDa, 7–8 nm), and scFv (~27 kDa) (Figure 6), and have a more stable and simpler structure [43]. The LOD of nanobody-based BioFETs has been proved to be sub-picomolar, which is at least five orders of magnitude lower than the previous reports of BioFETs based on aptamer [115].

Through comparison and analysis (Table 1), it is evident that the sensitivity of detection can vary significantly depending on the binding principle employed. These variations are further influenced by the methods used to immobilize the probes on the BioFET surface and the choice of transducing materials, highlighting the complex interplay between these factors. This observation underscores the role of transducing materials in dictating the overall performance of BioFETs. The following section will delve into the impact of transducing materials, discussing their properties, functionalization strategies, and implications for enhancing sensitivity and selectivity.

### 2.3. Transducing Materials

The transducing material plays a key role in BioFET performance, as it directly influences sensitivity, selectivity, and stability, which is critical for optimizing probe immobilization, preventing nonspecific adsorption, and reducing noise [46]. It is expected to drive significant improvements in device performance if designed properly [88]. Given that PD is a long-term therapy, the monitoring devices must prioritize long-term accuracy, efficiency, stability, and reusability to ensure reliable, ongoing patient care.

**Figure 7:**
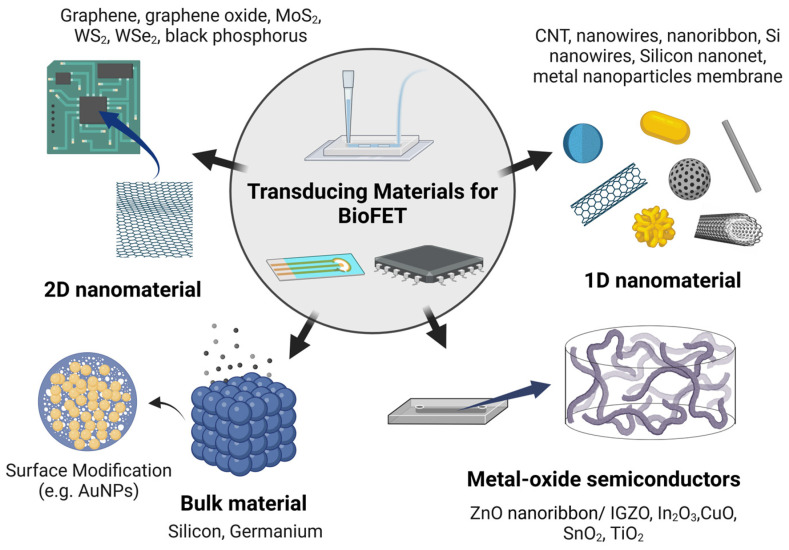
Common transducing materials used in BioFETs.

**Figure 8:**
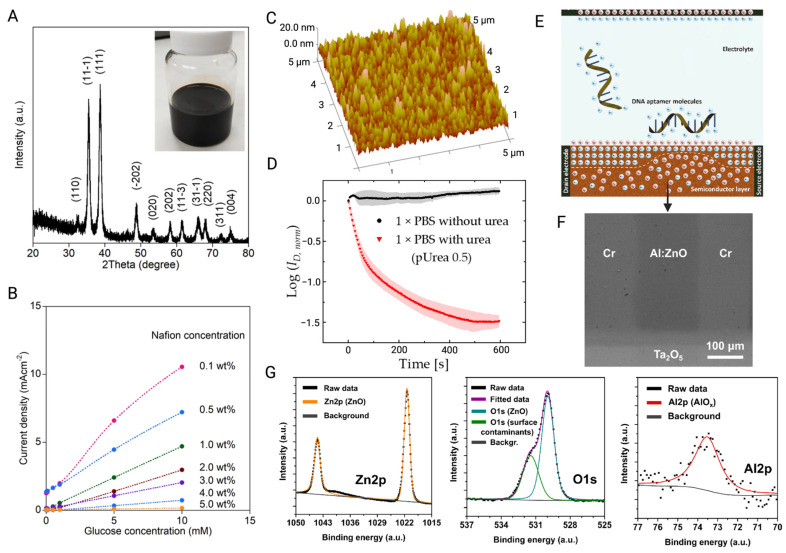
(**A**) XRD pattern of CuO NPs. The insert is a photo of CuO NPs ink. (**B**) The effect of Nafion coating concentration on responding to various glucose concentration at the 0.5 V potential [85]. (**C**) AFM analysis of Ag surface functionalized with urease. (**D**) The curve of *I_D_*/*I*_*D*0_ value when FET is exposed to 1 × PBS with and without target urea at a fixed *V_g_* of 0.3 V [139]. (**E**) Schematic of the principle of MOS-BioFET-based detection for DNA molecules. (**F**) SEM image of FET AI:ZnO channel. (**G**) XPS analysis of AI:ZnO film indicated the formation of AI-O-Zn bonds [140] (images used under Creative Commons licenses).

**Figure 9:**
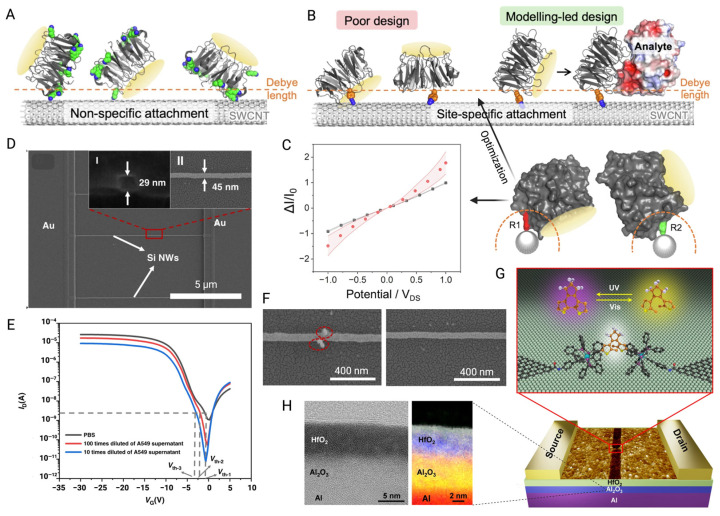
(**A**) The immobilization of the non-specific receptor protein (BLIP2) on the SWCNT channel of FET leads to binding events exceeding the Debye screening length. Green parts represent lysine residues. (**B**) Poor design part indicates that the binding site of BLIP2 is obstructed by the CNT or that the binding reaction occurs outside the Debye length. The proposed modeling-led design optimizes analyte binding within the Debye length and introduces an electrostatic profile to the SWCNT. (**C**) I-V_DS_ before (black) and after (red) the attachment of two viable rotamers for SWCNT docking. The shaded region is the standard error of the average values [141]. (**D**) SEM image of SiNW channel. Inset I and II are the SEM images of cross section and top view, respectively. (**E**) *I_ds_*-*V_gs_* curves of BioFET measured with various cell concentrations and PBS. (**F**) SEM image of exosomes captured by the CD63 antibody-modified FET channel and control channel [50]. (**G**) Schematic of single-molecule FET structure with a single dinuclear ruthenium-diarylethene (Ru-DAE) complex connects the nanogapped graphene point contacts for optimized functions. (**H**) Left: STEM analysis of the cross section of the Al/Al_2_O_3_/HfO_2_ multilayer structure under 200 kV. Right: XPS analysis of the dielectric layer with 5 nm Al_2_O_3_ and 5 nm HfO_2_ [142] (images used under Creative Commons licenses).

Transducing materials can be broadly categorized based on their dimensional structure (Figure 7); 3D bulk materials, such as silicon (Si) and germanium (Ge), are commonly used [134], as well as nanomaterials, such as metal oxide semiconductors (MOS), including zinc oxide (ZnO) and indium gallium zinc oxide (IGZO) (Figure 8) [135]. One-dimensional (1D) nanostructures, such as silicon wires (SiNWs) [50] and carbon nanotubes (CNTs) [86], along with two-dimensional (2D) nanomaterials, such as graphene [79], black phosphorus [136], or transition metal dichalcogenides (TMDCs), such as MoS_2_ [137], are also frequently employed (Figure 9). Additionally, metal nanoparticles are often used to modify the surfaces of these materials to amplify detection signals [138].

#### 2.3.1. Bulk Materials

Conventional FETs use 3D semiconductors such as Si/Ge as channel materials, typically with a thickness of 10^2^–10^4^ nm [143]. However, these materials are often seen as limiting in terms of signal conversion efficiency due to poor modulation of channels near the substrate [143]. While there have been efforts to enhance the sensitivity of BioFETs based on 3D materials by adjusting oxide thickness [134] or tuning the mole fraction in Si/Ge [144], they still present inherent limitations. Their bulkier structure results in weaker electrostatics and [145], although the increased surface area in 3D nanostructured electrodes can strengthen signal output, it also tends to introduce more noise [75,146]. In contrast, nanomaterials are far more appealing due to their high surface-to-volume ratio, superior electron transport properties, and sizes that are inherently compatible with biomolecules, etc. [147]. These attributes allow for the immobilization of a higher density of bioreceptors and provide more active binding sites, which can significantly improve the sensitivity and performance of BioFETs [138].

#### 2.3.2. Metal-Oxide Semiconductors

Compared to other materials, the main advantages of MOS are its ease of fabrication and compatibility with flexible substrates, especially in the field of wearable devices [148]. MOS, such as In_2_O_3_, ZnO, and SnO_2_, have wide bandgap characteristics, and their electrical properties are less affected by sensing environmental factors [149], which makes them competitive as the candidates of transducing materials in PD monitoring devices. They have been widely incorporated into BioFETs to develop PoC devices [149]. ZnO, which has a large and direct band gap (3.37 eV), can maintain a low noise level even under high pressure up to 200 kPa [150,151]. However, to enhance the stability of the system, high-k dielectric layers are frequently used to modify the MOS surface. The same passivation strategy works in other kinds of MOS as well, and the spin-coated Al_2_O_3_ layer on the surface of In_2_O_3_ BioFETs improved its insufficient stability in high voltage and liquid. The results show that the addition of the Al_2_O_3_ dielectric layer not only suppressed the damage of the In_2_O_3_ layer by leakage current and ion diffusion, but also exhibited long-term continuous monitoring performance with low signal drift [152].

Although MOS-based BioFETs exhibit high electrical performance, issues with mechanical flexibility usually limit their development in the field of wearable devices [153]. To fix this, it can be integrated with soft substrate to enhance flexibility. Studies showed that fabricating an array of MOS nanoribbon BioFETs on a polyethylene terephthalate (PET) substrate can maintain stable performance and high electron mobility even over 100 bending cycles, while it has a wide serotonin detection range spanning eight orders of magnitude [148]. A flexible printed circuit board (FPCB), which is more accessible, has been applied as well. In_2_O_3_ BioFETs were passivated with Al_2_O_3_ and the SU-8 layer and then incorporated into a FPCB to achieve multiplexing and simultaneous measurement of H^+^, Na^+^, and K^+^ concentrations. The FPCB-BioFETs devices exhibited strong stability and maintain ion monitoring for more than 90 days [153].

#### 2.3.3. One-Dimensional Nanomaterials

The 1D materials including SiNWs and CNTs offer a high surface area-to-volume ratio, with most of their atoms exposed to the surface. This feature brings them unprecedented sensitivity to the environmental changes [138], enabling detection at the single molecule level [154] or even down to single amino acid bases [155]. For instance, when CNTs are positioned near biomolecules, target molecules are attracted to the nanotube surface through covalent bonds or π–π interactions. These interactions cause the target molecules to fold or bind to the probes, altering the electrostatic potential (ESP) on the CNT surface [154]. The conductivity of these materials can be further enhanced through polymer coatings or improved deposition techniques. For example, loading polyethyleneimine (PEI) onto the CNT surface can create stronger binding [69], while optimizing deposition methods can improve the purity and yield of semiconductors [35], which in turn facilitates more efficient charge transfer. These strategies significantly enhance the performance of CNT-based BioFETs.

However, variations in the manufacturing of sensors can lead to inconsistencies in their electrical properties, making recalibration cumbersome and often unreliable, which hinders their commercialization and broader applicability [156]. One of the key advantages of 1D materials is their compatibility with the CMOS process, which allows for precise control over parameters such as length, width, density, and substrate doping level [126]. This compatibility enables large-scale manufacturing and integration [50], making 1D materials promising for future BioFETs applications. For instance, Zhao et al. [50] fabricated SiNWs BioFETs using a standard 8-inch CMOS processing platform, with nanowire dimensions of 45 nm in width and 10 μm in length. They used the APTES/glutaraldehyde method to immobilize CD63 antibodies on the surface of SiNWs for specific capture of exosomes by utilizing the properties of aldehyde and amino groups. They immobilized CD63 antibodies onto the SiNWs using the APTES/glutaraldehyde method, leveraging the aldehyde and amino group properties to specifically capture exosomes. The repeatability of the device was verified with a relative standard deviation (RSD) of 2.56% across three tests in exosome solution, indicating consistent device performance. Additionally, reusability was demonstrated by successfully separating captured exosomes using IgG elution buffer and reintroducing the exosome solution, with a threshold voltage change of less than 1.5. The standardized CMOS process not only minimized performance variation between devices, but also ensured good stability and reusability. These advantages make BioFETs more affordable, reliable, and suitable for long-term monitoring in PD patients.

The 1D materials show compatibility with smaller probes such as monoclonal antibody (Fab) fragments as well. These probes reduce the overall thickness of BioFET functionalization while it retains the binding sites and enhances the selectivity compared to antibodies, which makes the FET more resilient to Debye screening in high-ionic solution [126]. Studies have shown that Fab-based SiNWs BioFETs can achieve highly sensitive detection of CRP [126]. To further improve sensitivity, the surface of nanowires is optimized to have smooth edges, which increases mechanical stability and reduces electron scattering [157]. Techniques such as wet etching based on tetramethylammonium hydroxide (TMAH) and isopropyl alcohol (IPA) are employed to maintain the mask pattern and smooth edge defects [158]. However, research also demonstrated that increasing the surface roughness of SiNWs, by adjusting etching molecule concentration, can boost sensitivity. A rougher surface increases the surface-to-volume ratio, which enhances the system’s ability to capture target molecules more effectively, ultimately improving the BioFETs’ performance [159].

#### 2.3.4. Two-Dimensional Nanomaterials

Similar to 1D materials, 2D materials such as graphene and TDMC offer a high surface area and exceptional electron transport properties, while also being compatible with existing manufacturing processes. However, the cost of large-scale production for 1D nanomaterials is relatively high compared to 2D materials [57]. BioFETs based on 2D nanomaterials benefit from better control over their geometric properties, including minimizing impurities and crystal defects [160], as well as fine-tuning the band gap [147]. These advantages allow for more precise sensor performance and adaptability in various applications. Additionally, a 2D-based material channel can cause significant changes in Fermi level with its higher quantum capacitance [70]. This is important when detecting biomarkers in physiological fluids such as saliva, where concentrations are typically lower than in serum [161]. Achieving high sensitivity and LOD is crucial in these scenarios. As a result, BioFETs based on 2D materials are increasingly being widely utilized for detecting PD-related biomarkers and pathogens, offering improved performance in low-concentration environments [162].

Graphene, a single-atom-thick, tightly bound sp^2^-hybridized carbon layer [147], was the first discovered and utilized 2D nanomaterial [163]. Its unique electronic property—where the valence and conduction bands meet at a single point—makes graphene highly sensitive to external stimuli, a feature that is key to its use in biosensors. This sensitivity stems from its “fragile” band gap, allowing graphene to detect minute changes in its environment [147]. Furthermore, it remains the only 2D material with potential for large-scale commercial applications [163], largely due to its compatibility with chemical vapor deposition (CVD) technology [65]. This technique ensures the high-quality, cost-effective production of graphene with controllable size and thickness, making it suitable for mass production [164]. Monolayer graphene, in particular, preserves optimal carrier mobility, which is critical for the performance of graphene-based BioFETs [165]. The quality of graphene can be validated using Raman spectroscopy, where a 2.5:1 ratio of the G-band to the 2D-band and the absence of the D-band confirm its monolayer purity [166]. Compared to traditional silicon-based BioFETs, the operating voltage (VDS) required for these carbon-based materials is extremely low, reducing the risk of denaturing or degrading sensitive biological probes due to high voltage [138]. Because graphene’s almost nonexistent band gap and exceptional conductivity allow carriers to be easily generated and moved under an electric field [31]. This low VDS is beneficial for the development of portable and wearable BioFETs in the PD applications, as it extends battery life and makes these devices more user-friendly in real-world settings.

In addition to graphene, its derivatives—graphene oxide (GO) and reduced graphene oxide (rGO)—offer distinct advantages due to their high carrier concentration, light transmittance, chemical inertness, and biocompatibility [167]. The introduction of oxygen-containing functional groups in GO and rGO enhances their ability to form conductive films and facilitates molecular immobilization, which is crucial for sensor design [168]. Additionally, it can be used as a quencher for the fluorescent group to develop multiple sensing mechanism BioFETs. The oxygen-rich groups and excellent light transmittance of GO quench fluorescence from labeled aptamers, generating a measurable signal [169]. Compared to BioFETs employing a single sensing approach, dual-sensing BioFETs that integrate both electrical and optical detection methods are thought to offer improved reliability and detection accuracy [169]. However, while pristine graphene layers exhibit no D band, indicating a defect-free structure as shown by Kong, et al. [166], the presence of a D band in Zhang, et al. [169] suggested surface defects introduced during the transfer process. Interestingly, Kwon, et al. [170] demonstrated that intentional introduction of edge defects in graphene can enhance its sensing performance. These defects create stronger chemical adsorption sites, improving charge transfer at the probe–graphene interface, thereby boosting the sensitivity of the BioFETs.

While graphene’s zero bandgap provides high sensitivity, it also results in higher leakage currents, reducing the sensor’s dynamic range [160]. This issue arises because electrons can easily pass through the barrier in graphene, leading to increased leakage current and degraded sensor performance due to a higher subthreshold swing. In contrast, 2D materials such as MoS_2_, which have appropriate bandgaps, offer better alternatives for improving BioFET performance [147]. MoS_2_ is the most widely studied TMDC, which has a simpler structure than 1D materials [171]. Its thin atomic layer structures can reduce the short channel effect of BioFETs while maintaining high carrier mobility to provide excellent electrostatic effects [65]. Meanwhile, the planar architecture and exposed overhanging surfaces of MoS_2_ simplify its patterning as a semiconductor channel for BioFETs [147]. This makes it more suitable for high-precision applications, especially with nanoporous MoS_2_, which shows sensitivity up to 10⁹ times higher than pristine MoS_2_ in BioFETs sensing [172]. The bioreceptors can be coupled on the edge of MoS_2_ nanopores for specific signal generation. The edges of MoS_2_ nanopores can be functionalized with bioreceptors, which generate specific signals, and this edge-functionalization approach has been shown to induce larger conductivity changes in the device [173].

However, similar to graphene, MoS_2_ is susceptible to surface degradation, which can compromise the electrical reliability of BioFETs. To address this, MoS_2_ is often passivated with grafted layers such as Al_2_O_3_, TiO_2_, or gold nanoparticles. For example, depositing an ultrathin Al_2_O_3_ dielectric layer on nanoporous MoS_2_ helps prevent surface degradation when exposed to air or liquids, while also providing dangling bonds for the chemical adsorption of biomolecules [174]. However, the conflict is that this passivation increases the physical distance between the FET channel and the charged biomolecules, potentially reducing sensitivity. Therefore, careful control of the thickness of the passivation layer is critical to maintaining the balance between protection and sensitivity [172].

In sum, each material offers distinct advantages for BioFETs in detecting PD biomarkers, making it essential to consider the specific requirements of the application. Factors such as the electrical and mechanical stability of the measurement environment (i.e., PDE), sensitivity to light and temperature, material availability for scalable production (especially for PD PoC devices), and compatibility with flexible substrates [43] are crucial in selecting the optimal material. For example, 2D materials can provide higher sensitivity in some cases due to their larger surface area, improved electronic uniformity, and stability, making them well-suited for integration into flexible and wearable electronics, while 1D materials may excel in applications requiring high specificity and selectivity. Examples and omparison of these different transducing materials are summarized in Table 2.

### 2.4. Configuration

**Figure 10:**
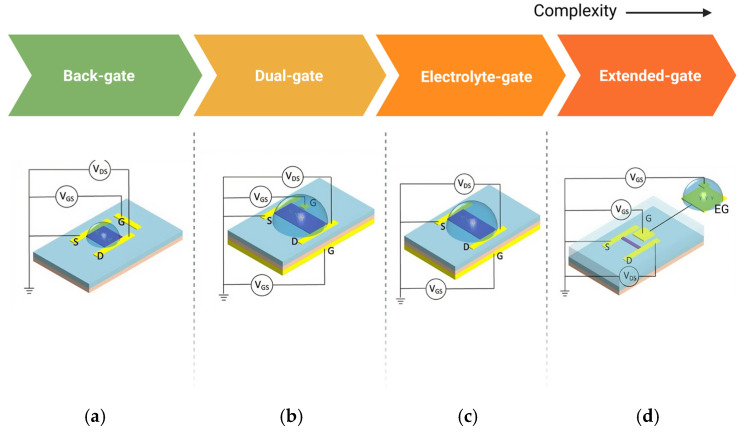
Various configurations of BioFETs. (**a**) back-gate; (**b**) dual-gate; (**c**) electrolyte-gate; and (**d**) extended-gate (edited from Nguyen et al. [43], under Creative Commons license).

The configuration of BioFETs plays a crucial role in influencing the sensor’s performance, even though the underlying principle remains the same across different types [182]. For example, in graphene-based FETs (GFETs), detection of bioreceptor–analyte interactions depends heavily on the electrostatic gating effects of graphene [163]. Based on how gate voltage is applied, BioFETs can be categorized into several configurations, including back-gated, dual-gate, electrolyte-gated (EGFETs), and extended-gate designs (Figure 10).

Each configuration offers distinct advantages and trade-offs for biosensing applications: Back-gated BioFETs typically involve a gate placed beneath the substrate, allowing basic electrostatic control, but may limit sensitivity due to the separation between the gate and the channel [183]. An example is the use of capacitive sensing applications, using Al as the back gate electrode and AlO_x_ formed by natural oxidation of Al as the insulating layer. The capacitance of the device proved to be well modulated by the gate voltage under vacuum conditions due to the quantum capacitance effect [184]. Dual-gate BioFETs, on the other hand, provide better sensitivity and control by using two gates to modulate the channel’s charge carrier concentration more effectively. For instance, Meng et al. [142] demonstrated a dual-gate single-molecule GFET with HfO_2_/Al_2_O_3_ as the dielectric layer, with nanocracked graphene electrodes and a single dinuclear ruthenium diene (Ru-DAE) complex as the channel, achieving a maximum on/off ratio of greater than three orders of magnitude. Extended-gate configurations decouple the sensing area from the active region of BioFETs, which helps reduce interference from direct exposure to the electrolyte, making it suitable for long-term and more durable sensor applications [101]. The limitation of EnFETs in combining several different active layers in one device also indicates the importance of extended-gate configuration [185]. For example, extending the gate through an externally connected platinum electrode functionalized with a graphene sheet decorated with bioreceptors can overcome the Debye screening limitation [186].

Compared to these gate configurations, electrolyte gate is the most common structure of GFETs [187]. Studies showed that electrolyte-gated configurations can be two orders of magnitude more efficient than traditional back gates [79] because the formation of a thin electrical double layer (EDL) between the electrolyte and the graphene interface improving device conductivity while ensuring low leakage current, and also the charge density in these devices depends more on the thickness of the EDL rather than the distance between the gate and channel [188]. In electrolyte gate GFETs, the sample solution is directly applied as the electrolyte or proxy of physical fluid and bioreceptors are fixed on the graphene surface to capture the targets [189]. However, such devices often need an additional passivation layer to avoid contact between the source and drain electrodes through the electrolyte solution to ensure the stable contact with the analyte [167]. Overall, each of these configurations affects the overall sensitivity, stability, and selectivity of the BioFETs, and the choice of configuration depends on the specific application requirements.

## 3. Functions of BioFETs Applicable to Peritoneal Dialysis Monitoring

Continuous monitoring of key substances such as urea, creatinine, and electrolytes (H^+^, K^+^, and Na^+^) is essential for assessing dialysis status (Table 1) [190]. They can serve as critical risk assessment tools for identifying patients at the highest risk of developing PD-related complications, facilitating personalized treatment interventions [191]. For instance, biomarkers that indicate peritoneal inflammation can help identify patients with progressive loss of peritoneal function, while those reflecting weakened immunity can identify PD patients with increased susceptibility. Shifts in cytokine patterns may provide insights into the nature of an infection [191]. However, most of the current methods of monitoring PD patients are limited to delivering doses and measuring the transport status of the peritoneal membranes, and the medical requirements for biomarkers are still unmet [10,191]. The integration of BioFETs into PD monitoring systems has the potential to enable sensitive, real-time and PoC analysis of PDE composition, helping to identify complications including peritonitis, membrane failure, or fibrosis [192] at an early stage. In this section, we explore the potential application scenarios of BioFETs applicable to PD monitoring by dividing their functions into routine monitoring and biomarker detection for early diagnosis and intervention, highlighting their significance in improving BioFET management.

### 3.1. Routine Monitoring of Key Indicators

#### 3.1.1. Dynamic Monitoring of Glucose Imbalance

In PD, the glucose concentration in the dialysate is typically 10 to 50 times higher than physiological serum levels, creating an osmotic gradient essential for effective fluid removal [193]. However, elevated glucose levels contribute to complications such as hypertriglyceridemia, hyperglycemia [15], structural and functional changes in the peritoneum [20], and, eventually, fibrosis, either directly or indirectly, through the formation and adsorption of glucose degradation products (GDPs) and advanced glycation end-products (AGEs) [194]. Although various alternative therapies, such as stem cells [195] and peptides or glucose polymers [196], and macromolecular solutions such as L-carnitine and xylitol [193], have been proposed, none gained widespread acceptance. Consequently, glucose remains the predominant osmotic agent due to its effectiveness, low cost, and favorable safety profile [58]. So, the latest Kidney Disease Improving Global Outcomes (KDIGO) guidelines recommend continuous glucose monitoring (CGM) for ESRD patients [197]. However, recent CGM studies focus on hemodialysis, with relatively little attention to blood glucose self-monitoring (SMBG) [197]. It is, therefore, important to offer PD patients accessible methods for self-monitoring glucose levels to enable personalized treatment adjustment, especially since absorption varies depending on individual peritoneal characteristics and transporter status [198].

BioFETs-based glucose monitoring operates through an enzymatic reaction between glucose oxidase (GOD) and glucose [167]. In this process, glucose is catalyzed by GOD to produce gluconate and H_2_O_2_, where the H_2_O_2_ goes on to be dissociated and produces H^+^, which can be detected by the BioFETs [199]. More specifically, H_2_O_2_ undergoes electrocatalytic oxidation at a microelectrode to generate H_3_O^+^, and the subsequent diffusion-mediated transport of hydronium ions creates localized pH gradients detectable by BioFETs [200]. However, as mentioned above, enzyme-based BioFETs are intolerant to challenges such as long-term use and storage. The immobilization efficiency of enzymes on BioFETs can be compromised by factors such as ambient dissolved oxygen levels, temperature, and humidity fluctuations [68], resulting in lower dissociation constants for the gluconic acid and a limited dynamic range in practical applications [200].

To eradicate the limitation of enzyme-based BioFETs, research shifted toward developing non-enzymatic BioFETs. One approach involves depositing CuO NPs in the channel of an EGFET using a low-cost inkjet printing technique. The catalytic properties of CuO NPs influence the glucose oxidation process, with the oxidation peak current increasing as glucose concentration rises, enabling quantitative glucose analysis [85]. However, these BioFETs suffer from poor selectivity toward different sugars. Recent studies demonstrated that transition metal oxide-based BioFETs offered improved electrocatalytic activity and significantly enhanced glucose oxidation, particularly in compound in the form of MWO_4_ (M could be Ni, Cu, etc.). Shaping these materials into porous microspheres further increased their surface-to-volume ratio, enhancing sensitivity. Notably, NiWO_4_-based BioFETs retained good selectivity for glucose in the presence of interfering substances such as ascorbic acid, uric acid, lactose, fructose, and dopamine [68]. These advancements present promising potential for non-invasive glucose detection in next-generation biosensors.

Efforts to introduce more stable glucose-sensing components and improved immobilization strategies have also been explored to enhance FET-based glucose detection. For instance, modifying the rGO surface with AuNPs can significantly boost the electro-oxidation of glucose, resulting in a more stable structure with a wider linear detection range compared to EnFETs. Notably, this configuration achieves a LOD for glucose that is nearly ten times lower than traditional electrochemical sensors [201]. Optimizing the GOD immobilization further contributes to better FET performance. Compounds such as glutaraldehyde and Nafion have been employed to strengthen the adhesion of GOD to the channel. Additionally, incorporating a TiO_2_ film into the system enhances sensitivity at extremely low glucose concentrations [202]. TiO_2_’s photoelectrochemical properties make it particularly promising for glucose detection, with ongoing research aimed at increasing its light absorption efficiency. This is achieved through heterojunctions with other carbon or gold nanomaterials, as they need to be sensitive enough to detect trace glucose levels in, for example, saliva and sweat [203]. This heterojunction method not only increases the signal strength, but also improves the stability of the sensor, which may facilitate glucose detection in other human fluids for PD patients.

However, the biocompatibility of BioFETs material needs to be considered to hinder adverse clinical reactions [204]. For example, polymeric ion-sensitive membranes (ISMs), which are often conjugated into BioFETs, may involve plasticizers with possible cytotoxicity [75]. To address these concerns, more biocompatible materials, such as vinylphenylphenylboronic acid (VPBA), have been explored as glucose-responsive monomers. Combined with 2-hydroxyethyl methacrylate [160], these can form a functionalized hydrogel coating on the BioFETs electrodes for glucose monitoring [205]. The hydrogel-based BioFETs offer high sensitivity and biocompatibility, making them suitable for both in vivo glucose monitoring and in vitro applications, including wearable devices such as glucose-sensing contact lenses [205]. The development of these devices aligns with the needs of PD for daily glucose monitoring, particularly as they can be adapted into PoC systems equipped with wireless capabilities, enabling non-invasive and wearable biomarker detection for both patents and clinicians [75].

In a similar vein, hydrogels can encapsulate other glucose-sensitive parts, such as boronic acid-containing peptides, in BioFETs. These peptides can locally self-assemble into a hydrogel nanofiber network through non-covalent interactions, where phenylpropionic acid interacts reversibly with glucose, producing borate ions that influence the conductivity of the semiconductor [59]. These hydrogel-gated BioFETs demonstrate potential for wearable applications, allowing real-time monitoring of glucose in complex environments. It also proves that enclosing interfacial hydrogels on BioFETs may be a general strategy to overcome the stability issues of wearable BioFETs. Furthermore, enhancing the reusability of non-enzymatic BioFETs is possible by using sensing parts such as N-dimethylaminopropyl acrylamide, which undergoes reversible reactions with the glucose. Studies have shown that such modified BioFETs exhibited a glucose LOD of 1.9 μM in human urine, and they can regain the detection ability after hydrochloric acid treatment [178].

#### 3.1.2. Urea and Creatinine Clearance

Routine monitoring of urea clearance (Kt/V) and creatinine clearance (CCr) is a clinical standard for assessing residual renal function [206,207]. Both urea and creatinine are filtered by the glomerulus, but urea will be reabsorbed while creatinine will be excreted. Therefore, evaluating urea or creatinine clearance alone may misjudge the residual renal function [44]. This makes the urea-to-creatinine ratio (UCR) particularly valuable in clinical practice, as an elevated UCR typically signals declining renal function [208], while their concentrations in PDE are commonly used to assess the clearance of toxic molecules during dialysis [209]. According to the International Society for Peritoneal Dialysis (ISPD), current dialysis prescriptions are adjusted based on the proportion of urea removed from the body [210]. Urea concentration during PD should be calculated through the weekly total kt/V [211]. The dimensionless parameter Kt/V represents the dialysis level, where K is the urea clearance, t is the duration of the treatment and V is the body urea distribution volume. It is usually measured before and after treatment, or indirectly by the conductivity of the dialysate [55].

Spectrophotometry has long been a standard laboratory technique for urea detection [212]. However, its high cost and infrastructure requirements make it unsuitable for real-time monitoring. Moreover, to obtain an accurate Kt/V, samples ideally need to be taken 30 min after treatment to account for the urea rebound effect, which cannot be captured without continuous monitoring, leading to potential inaccuracies [213]. While urease-based urea sensors were once popular due to the enzyme’s low cost, availability, and stability [214], they faced significant drawbacks. These sensors rely on the hydrolysis of urea by urease, producing hydroxyl ions after being catalyzed by urease, leading to a change in pH so that the urea concentration can be obtained by measuring the change in hydroxyl ion concentration [215]. However, challenges such as low selectivity, frequent calibration, and maintenance issues hindered their widespread adoption [213]. To avoid unnecessary delays during the treatment, urea concentration in PDE should be better measured under flow conditions [216] which poses high pressure on the sensitivity of sensors.

BioFETs, by contrast, offer significant potential for real-time urea detection in PD systems. EnFETs can detect pH changes or dissociated NH_4_^+^ and HCO^3−^ generated by urease-catalyzed reactions, converting these into electronic signals proportional to urea concentration [217]. Early designs of BioFETs for urea detection utilized pH-sensitive ion-selective BioFETs (ISFETs), where urease was immobilized on a hydrated silicon nitride surface using glutaraldehyde [218]. However, the small sensing area of traditional BioFETs limited the enzyme density, resulting in weaker output signals [139]. To allow for a larger sensing area, EGFETs were developed, where the gate electrode is positioned away from the BioFETs, preventing direct contact with the target analyte [101]. This design not only expanded the sensing area, but also improved immobilization efficiency and sensitivity. In the case of urea detection, urease was immobilized on membranes and applied to the extended gates of ISFETs to create PoC devices, enhancing sensitivity by up to 50 times compared to earlier urea detection systems [219]. The setup can be cost-effective by directly fixing urease on commercially available fluorine-doped thin oxides (SnO_2_:F), yielding stable results within one minute using these simple assembly BioFETs [214]. Another approach involves embedding urease in magnetic alginate microcubes, which are immobilized on the EGFET surface via an external magnet. This straightforward packaging technique is also applicable to other substances, such as antibodies. By integrating different functionalized magnetic beads into a microfluidic chip, multiple biomarkers, such as urea, glucose, and proteins, can be monitored simultaneously in real time [215].

However, EGFETs have limitations as mentioned above, such as the inherent interface between the gate and the membrane, which introduces additional parasitic capacitance and resistance, negatively impacting conductivity and reproducibility [139]. To maintain the sensitivity and usability of BioFETs in real human fluids, urease was directly fixed on the Ag gate of the EGFET. This modification allowed for higher receptor densities, significantly enhancing the output signal. The device exhibits very low extraction power consumption compared to other conventional BioFETs, making it more applicable for practical applications [139].

Creatinine is also one of the most widely used biomarkers for assessing kidney function, though it should be accompanied with other biomarkers to optimize diagnosis or indicate the kidney status [220]. The conventional method for detecting creatinine in clinical settings is the Jaffé method [221], but this approach lacks specificity and a timely response, especially in the presence of interfering substances found in biological fluids [222]. Low accuracy, toxicity, and non-specific adhesion of ammonium ions used in this system limited this method as well [223]. To address these limitations, modern electrochemical biosensors have been developed to offer a more sensitive and specific interface for creatinine detection, utilizing biological receptors such as enzymes or novel synthetic responsive materials [224]. However, enzymatic systems are the most reported electrochemical creatinine sensors, prized for their high selectivity [225], and enzyme layers have been thoroughly investigated and applied in BioFET development [226].

EnFETs have been employed as potentiometric methods for creatinine detection, with creatinine deiminase [227] immobilized on the carboxyl-functionalized multi-walled carbon nanotube (COOH-MWCNT) films through crosslinking techniques. This setup, achieved through a chemical solution method, does not require complex instruments [228]. The strong bonding between COOH-MWCNT and CD over a large area improves the conductivity of the biosensitive membrane. However, while the crosslinking technique enhances stability, it is thought to reduce sensitivity and analytical range [224]. Moreover, despite improved assay efficiency [223], these enzymes still suffer from stability issues and are costly to apply in practice, as previously mentioned [229].

To improve the reliability of EnFETs for creatinine detection, special consideration should be given to issues related to enzyme handling, storage, or aging. Researchers explored various strategies to address these shortcomings, including immobilizing enzymes with nanoparticles to increase surface area and facilitate higher charge transfer [225], as well as improving CD adsorption [230]. One approach involved immobilizing CD on silicate particles, which were then coated onto the surface of a pH-sensitive BioFET for creatinine detection in physiological solutions. This design demonstrated high signal reproducibility and stability, with the device functions remaining for over a year in storage. Compared with traditional enzyme covalent crosslinking through glutaraldehyde vapor, this technique provided two- to three-fold increases in creatinine sensitivity, three- to four-fold reductions in response and recovery times, and significantly lower assay thresholds [231]. Adsorption of CD onto zeolites has also proven to be a non-toxic and stable method to retain enzyme activity. The effect of different modified zeolites on the BioFETs-based detection response has been investigated, with the zeolite modified with AuNPs (BEA-Gold) showing the highest sensitivity for creatinine detection. The incorporation of gold nanoparticles not only helped prevent enzyme denaturation, but also increased the surface area [230]. This aligns with early studies, since the nitrogen on the aromatic ring of creatinine exhibits a strong affinity for metal ions, making metal nanomaterials especially effective for creatinine detection [225]. In fact, several reviews discussed creatinine detection methods using different metal-centered nanomaterials [232], further emphasizing their suitability for this application.

Despite these improvements, enzymes remain costly and susceptible to denaturation over time [225]. Although studies on non-enzymatic BioFETs are less extensive than their enzymatic counterparts, these devices can be tailored to meet specific sensing requirements and are simpler to fabricate [232]. One of the most studied non-enzymatic approaches involves the use of molecularly imprinted polymers (MIPs), which are often used to replace the gate ends in ion-sensitive BioFETs, creating a sensor that responds specifically to creatinine-sensitive ions [223]. MIPs are artificially synthesized receptors that mimic the mechanism of antibody–antigen formation with high affinity interactions [232]. In this method, templates and cavities are created in a high-affinity polymer matrix [223], and MIPs are imprinted onto elongated BioFET electrodes, reporting creatinine concentrations through integration with a digital readout circuit ring oscillator [233]. To improve the sensitivity of these systems, optimizing MIP-related parameters is crucial for improving the conductivity of BioFETs, a topic that will be explored in further sections.

### 3.2. Detection of Potential Biomarkers

In addition to daily monitoring functions for glucose, creatinine, and urea, BioFETs have shown significant potential for detecting a wide range of biomarkers. These include PD-relevant biomarkers such as cystatin C (CysC), beta-2 microglobulin, albumin [234], cell-free nucleic acids, microRNA [235], inflammation markers such as interleukin-6, tumor necrosis factor-alpha (TNF-α) [236], and exosomes [237], among others (Table 3). PDE is a rich source of these potential biomarkers [238], containing solutes diffusing from the circulation along with peptides and proteins released locally from the peritoneal tissues. These components can provide insights into mesothelial cell mass, peritoneal fibrosis, and local inflammation, to name a few, during PD [239]. Additionally, other biological fluids, such as saliva, tears, sweat, serum, plasma, and urine, have also been explored as sources for PD biomarkers [240,241,242]. However, despite promising findings, the integration of new PDE biomarkers into routine PD monitoring remains limited while there is no proof that these biomarkers directly contribute to the PD outcomes [58]. The only two identified biomarkers so far are CA125 and IL-6, both of which can be readily measured in unconcentrated PDE [58,237].

#### 3.2.1. IL-6

IL-6 is the most thoroughly studied marker of inflammation in PD patients, and its systemic levels of IL-6 and its soluble receptor are always elevated in ESRD patients while inducing hepatic acute phase protein synthesis [191,239]. Additionally, it is a key factor in increasing solute transport associated with inflammation, which predisposes the peritoneum to fibrosis [254]. BioFETs-based IL-6 detection primarily leverages the highly specific interaction between IL-6 and its antibody, IL-6R [255]. When IL-6R is immobilized on transducing materials such as SWCNTs, it captures IL-6, causing a measurable drain current change in the BioFETs due to the antigen–antibody interaction. Simultaneously, inter-tube contact between the SWCNTs and the FET reduces resistance, further improving the detection sensitivity [255].

Efforts have been made to transform this system into a wearable PoC platform. For instance, IL-6 antibody was immobilized on the rGO layer and transferred to a flexible polycarbonate substrate to develop BioFETs, facilitating the ultra-sensitive IL-6 detection even in tears [256]. The IL-6 detection has been expanded to saliva as a non-invasive and accessible pathway as well. Aptamer-functionalized GFETs were integrated onto PCBs to form a portable PoC device for real-time monitoring of IL-6 in saliva. This system can respond to the changes in IL-6 concentration within several minutes and display results via an online app, making it a promising tool for the self-monitoring of PD patient users [187].

To increase the detection range of IL-6, a novel metal carbide nanocomposite MXene film with an accordion multilayer structure was applied to amplify the electrical signals. An IL-6 aptamer was also decorated with thiolate group at its 3’ end to improve the efficacy. Due to the combination of the optimized BioFET structure with the multi-helix structure, the BioFETs demonstrated an IL-6 detection range nearly two orders of magnitude higher than the previous one with better selectivity, reproducibility, and stability [257]. Despite the advancements in detecting IL-6 using BioFETs, its role as a definitive marker for peritoneal inflammation remains debated. IL-6 has been shown to downregulate other inflammatory molecules by activating IL-1 and TNF receptor antagonists, suggesting it also possesses anti-inflammatory properties. This dual functionality complicates its use as a straightforward indicator of peritoneal inflammation, as IL-6 may contribute to preserving the peritoneum rather than solely indicating inflammation [258].

#### 3.2.2. CA125

CA125, another identified substance in the PDE regular monitoring, is produced by cells lining the peritoneum and so is often used to reflect the quality of mesothelial cells of PD patients as an alternative parameter for determining the status of the peritoneum [259]. A sudden drop in CA125 levels may signal severe mesothelial cell damage [239] or a heightened risk of encapsulating peritoneal sclerosis [133] development [260]. However, elevated CA125 levels in the PDE may also occur during PD-related peritonitis [259], making CA125 monitoring essential for accurately diagnosing and evaluating the overall status of PD patients.

Relatively few studies explored CA125 detection using BioFETs compared to IL-6. A previous study immobilized CA125 ssDNA [261] on the surface of carboxylated MWCNT-BioFETs via amide bonding and then stacked it on the graphene surface of rGO-based BioFETs via π–π interactions. Through specific interactions with CA125 antigens, the BioFETs were capable of detecting CA125 with high specificity and a LOD of 5.0 × 10^−10^ U/mL, possessing the potential for application in biological fluids and clinical samples. To ensure BioFETs have high electrical performance and degradation stability in physical solutions for practical CA125 application, they were integrated with microfluidic channels using the liquid channel as a passivation layer, which effectively improves the electrical stability of BioFETs [262].

However, the routine measurement of peritoneal CA125 is not widely recommended [263] due to ongoing debates about its reliability as a biomarker, similar to IL-6. Additionally, CA125 levels are positively correlated with IL-6 [258]. Moreover, some CA125 in the PDE may be partially derived from systemic circulation [263], while the ideal PDE biomarker should be released or produced locally in the peritoneal cavity or be involved in the pathology of the peritoneum [239]. Furthermore, studies have shown no significant impact of peritoneal urea clearance or residual renal function on serum CA125 levels [264]. Consequently, CA125 is generally regarded as an auxiliary biomarker, often used in conjunction with other markers, such as Chemokine CCL2, to provide a more comprehensive understanding of the inflammatory process [265].

#### 3.2.3. Other Potential Biomarkers and Pathogens

The controversies surrounding IL-6 and CA125 have driven the development of new biomarkers, including various cytokines and growth factors such as IL-8 and IL-17 [58], many of which are soluble, making them readily accessible in human fluids [237]. PoC diagnosis devices have been developed for TNF-α detection in tears, where the TNF-α aptamer was directly immobilized on GFETs. A nonionic surfactant was used to minimize nonspecific binding, subsequently enhancing the specificity [266]. Similarly, CNT-based BioFETs designed for CRP detection had been reported, where CRP antibodies were immobilized, enabling the detection of CRP levels from normal to inflammatory ranges, showing promise for clinical use [35]. To further improve the sensitivity, CRP Fab fragments were used in place of full antibodies, addressing the Debye screening effect and reducing the overall thickness of the capture layer. These Fab fragments were incorporated into SiNW array BioFETs, which provided a larger surface area and more binding sites to accommodate more analytes, thereby expanding the detectable concentration range [250]. However, it is important to note that the possibility of systemic production of these biomarkers cannot be entirely ruled out. For example, VEGF and TNF-α detected in the PDE may also originate from systemic circulation [258].

In addition to the biomarkers mentioned, germ infections are common during PD due to prolonged catheterization [15], yet relatively few studies directly applied BioFETs to detect pathogens in the context of PD diagnosis [56]. While some research summarized the use of various FETs for pathogen detection, these studies largely focused on applications related to food safety and environment monitoring [267]. In the context of PD, BioFETs-based detection mainly targeted bacterial biofilms [56], cell wall proteins [268], and genetic material such as DNA or RNA [94]. For instance, fibronectin, a protein that binds specifically to certain bacterial components, has been fixed on Au-EGFETs to adhere to the Staphylococcus epidermidis (*S. epidermidis*) biofilm, leading to changes in conductivity. In the same study, ssDNA probes were used to target the 16S rRNA of Staphylococcus Aureus (*S. aureus*) and *S. epidermidis*, resulting in pathogen-specific detection [56]. Another noble study employed Au nanoporous membranes in combination with an extended-gate FET to hybridize with *S. aureus* 16S rRNA, demonstrating high specificity even in the presence of non-specific DNA from other pathogens. The nanoporous gold membrane, by providing a large surface area on both its surface and walls to attach probes, enhanced the hybridization signal. This enables better control over DNA orientation and immobilization, making further boost detection accurate [269]. Additionally, innovative research utilized DNA origami techniques to design RNA-cleaving deoxyribonuclease (RCD) specific to *S. aureus*, capable of detecting concentrations ranging from 1 to 10^5^ CFU/mL, with a remarkable lower LOD of 1 CFU/mL [270]. While the treatment approaches for Gram-positive (GPB) and Gram-negative bacteria (GNB) infections in PD are distinct [23], distinguishing bacterial types is crucial for effective initial antibiotic therapy. Recent advancements led to the development of BioFETs-based platforms designed specifically for this purpose. In these systems, antibiotics such as vancomycin, targeting GPB, and magainin I, targeting GNB, were immobilized on GFETs. These GFETs were subsequently integrated into a dual-channel microfluidic chip, enabling the simultaneous detection of multiple pathogens during PD treatment [268].

The ongoing development of these BioFETs is significant, as it enhances the capability to diagnose early infections in PD patients, thereby facilitating timely and appropriate treatment. Current examples and applications of using BioFETs to monitor these potential biomarkers related to PD are summarized in Table 4.

## 4. Challenges and Strategies for Implementation of BioFETs in PD Monitoring

### 4.1. Technical Barriers and Clinical Applicability

In practical applications, the complex composition of the PDE usually biases the detection and quantification, which poses a challenge for daily monitoring. The plasma-like structure of PDE, for instance, contains high-abundance proteins that interfere with proteomic analyses, masking signals from target biomarkers [238]. For other potential diagnostic samples, such as saliva, sweat, and tears, the concentration of target biomarkers is often considerably lower, posing additional hurdles for sensitive and accurate detection [281]. Moreover, to ensure reliable detection of analytes such as urea, measurement often requires ambulatory conditions [216]. These challenges are further exacerbated by the high-ionic concentrations in human bodily fluids, which induce Debye screening effects. This phenomenon involves an ionic cloud surrounding the target charge, diminishing its influence on the surface potential of the BioFETs gate. Consequently, the target charge can be significantly screened, leading to a substantial reduction in signal strength, potentially rendering the detection signal undetectable [71]. This interplay of complex fluid composition and stringent sensitivity and specificity demands underscores the significant technical barriers BioFETs face in PD monitoring applications.

In clinical applications, PDE samples could significantly heighten the risk of degradation for the transducing materials of BioFETs. These samples can vary widely in appearance and composition due to patient-specific conditions—for instance, red from peritoneal bleeding, milky due to triglyceride or chylomicron accumulation, yellow-green from bile leakage, or brown-black from hemolysis [282]. Such variations will make BioFETs channel prone to fouling, oxidation, and delamination over time [174]. Therefore, stability is paramount to ensure the reliable performance of BioFETs during prolonged monitoring in clinical settings, yet achieving this stability amidst the diverse and dynamic composition of clinical PDE samples remains a significant challenge.

Translating BioFETs from laboratory prototypes to commercially viable clinical tools poses significant scalability challenges. While current nanofabrication techniques are effective for small-scale production, they frequently fall short in providing the precision and reproducibility required for large-scale manufacturing processes. For example, most BioFETs built with 2D materials are only performed on a laboratory scale and not allowed for reproducible high-throughput manufacturing processes [57], because the non-uniform morphology of the substrate and the scattering effect can make the output of each device unreliable [147], but fortunately, advances such as standardized CMOS processes [50,126], compatibility with large-scale CVD [65], and lower-cost fabrication techniques including spray gun coating [112], inkjet printing [85], and reversible reaction designs [178] covered above, offer promising pathways to address these issues and promote commercial potential. However, integrating multiple probes into a single BioFET chip further increases manufacturing complexity, requiring highly specialized processes that are not yet widely available.

In addition to the consideration for BioFETs themselves, for BioFETs being adopted in clinical practice, they must integrate seamlessly into existing PD monitoring frameworks. This requires compatibility with standard clinical tools and protocols to ensure that BioFETs can be effectively used alongside current diagnostic and monitoring platforms. For instance, wearable BioFETs continuously monitoring inflammatory markers could trigger an alert if levels exceed a pre-defined threshold. This would prompt the clinicians to collect PDE samples for the further identification of peritonitis and initiation of targeted antibiotic therapy. This integrated approach could significantly reduce the time to diagnosis compared to relying solely on intermittent PDE analysis. Alternatively, a BioFETs-based patch could be connected to the catheter for the regular monitoring of solute clearance to personalize dialysis prescriptions, supplementing the information obtained from PET scans. By tracking trends in solute clearance over time, clinicians could identify patients who are experiencing a decline in peritoneal membrane function and adjust their dialysis regimen accordingly. Lastly, the data generated by these BioFETs can be transmitted via IoT devices (e.g., smartphones, tablets) to a secure cloud platform (Figure 2). This enables integration with existing patient records and facilitates clinical decision making. New algorithms or decision support tools may be required to effectively interpret the BioFETs data in conjunction with other clinical parameters, a topic that will be discussed in more detail subsequently. This integrated approach allows for a shift from traditional, clinician-centered monitoring towards a more patient-centered model, empowering individuals to actively participate in their PD management.

### 4.2. Solutions to Overcome Technical Barriers

#### 4.2.1. Enhancing Sensitivity and Selectivity

**Figure 11:**
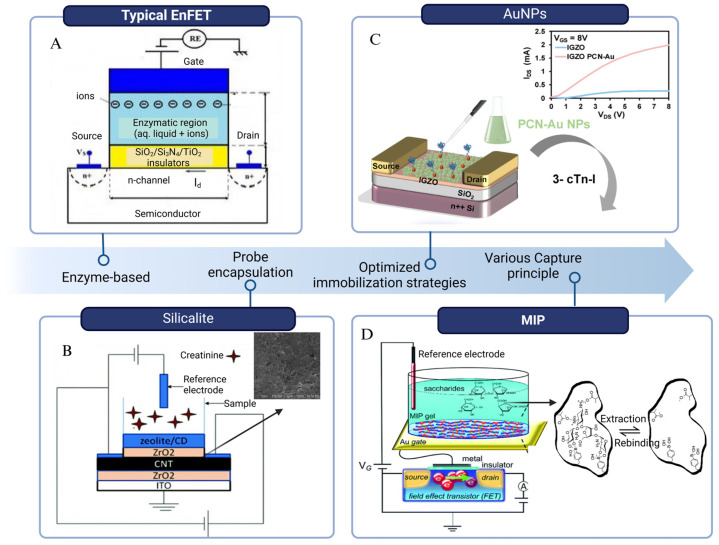
Schematic of some strategies for enhancing sensitivity. (**A**) A general model of EnFET [285]. (**B**) Silicalite was used as adsorbent at the top for the creatinine deiminase immobilization [69]. (**C**) Schematic diagram of the PCN-Au NPs IGZO TFT aptamer sensor, in which the aptamer is immobilized on Au NPs. The inset shows that under a constant bias voltage, the output characteristics of the PCN-Au NPs-modified IGZO are greatly optimized [135]. (**D**) Schematic of MIP-BioFET structure and illustration of MIP binding principle with paromomycin template as an example [286] (edited from images under Creative Commons licenses).

Efforts to enhance the sensitivity and selectivity of BioFETs for practical applications face challenges due to the high ionic strength of physiological environments and the complexity of sample compositions, and in particular, efforts to overcome the latter impairment are still very limited [62]. In recent years, pairwise efforts focused on new BioFETs designed to address these challenges, including the introduction of new materials [49], development of controlled immobilization methods, and optimization of probe designs (Figure 11) [62]. Additional innovations in fabrication architectures aim to reduce internal electrical noise [283] and extend the Debye screening length in solutions [284], advancing the integration potential of BioFETs within PD monitoring systems.

Appropriate immobilization strategies are crucial for achieving the good performance of BioFETs. For example, in urea detection, the immobilization efficiency of urease would have a large effect on the sensitivity and response time of the urea biosensor by increasing the hydrolysis rate of urea [287]. The immobilization step was thoroughly investigated to improve its stability and reduce signal interference, including enzyme encapsulation in alginate or hydrogel beads, gate surface modification with gold or magnetic nanoparticles [87], incorporation of the enzyme into polyelectrolyte multilayers such as polyaniline or Nafion [232] or silicalite [69], and nano-spotting technique [288]. These methods do not usually aid the enzymes directly, but rather serve as an enzyme loading matrix [86]. Alginate microbeads carrying enzymes can be fixed to the BioFET surface by an external magnetic field so that the analyte sample can be injected into the microchannel and react with the enzymes contained in the alginate beads. The potential change in the BioFET surface caused by the release of H^+^ during the reaction can indicate the glucose, urea, and creatinine levels, respectively [289]. A major advantage of this encapsulation method is that a single sensor chip can detect multiple analytes simultaneously and the enzyme depletion can be countered by easily replacing the alginate microbeads with a new one [290]. However, a comparison with the results of the commercial assay kits revealed that the sensitivity of this kind of device was not substantially increased compared to the sensitivity previously obtained using the conventional bulk alginate enzyme encapsulation method [290]. The differences between the two sets of results for the glucose, urea and, creatinine metrics were 5.17%, 6.22%, and 13.53%, respectively. This result might be attributed to the partial depletion of the enzyme in the microbeads and absorption of the biological sample by the surface of the microchannels, resulting in a slight decrease in the intensity of the response during the measurement [289]. This could be avoided if the enzyme was properly stabilized by selecting a more suitable substrate and better immobilization methods [291]. Despite the excellent sensitivity, other researchers argued that the use of such biomaterials is problematic due to the absence of enzymes or antibodies for each target biomarker, as well as for some other reasons, such as poor long-term stability, costly and time-consuming production, and difficulty in controlling the quality of the production, and that therefore there is a need to look for other alternatives with high sensitivity [75].

Au alone is usually considered to have no specificity and selectivity when used as an electrode film coating [75], but AuNPs are widely used for doping modification of carbon materials due to their high catalytic efficiency, good biocompatibility and chemical stability [30]. In creatinine detection, creatinine can be physically adsorbed on the active sites of various metal centers to form metal–creatinine complexes, and then the current signal changes [232]. The same principle has been applied to the detection of CRP. AuNPs acted as a defect marker for the MoS_2_ layer of FET and non-covalently binding the defected MoS_2_ edges. The formation of strong S-Au bonds improves the charge transfer in FET while the immobilization of the CRP antibody enabled the detection of CRP [137]. Recently, AuNPs were employed for the detection of cystatin C in urine samples for the CKD patients, which is likewise a promising biomarker for early detection of renal injury [223]. In this system, AuNPs not only served as a carrier for papain, which can selectively bind to cystatin C to form a complex, but also improved the surface chemical inertness of laser-induced graphene, eliminating the need for an additional surface activation step [30]. Despite offering so many benefits, it is not suitable for large-scale manufacturing and practical application due to the high cost of raw materials [292], while clinically more cost-effective tools are preferred.

Artificial and functional membranes are quite promising, serving as platforms for small biomarker molecular recognition sites [72]. Molecularly imprinted polymers (MIPs) can not only overcome the above-mentioned biomaterial problems, but also enhance the binding constants (K_a_) and LOD [75]. The response principle of MIPs to the target substance is that the chemical reaction between the template and the molecule generates charge, or the template molecule is embedded in the template, resulting in a weakened charge transfer to the electrode surface [275]. These polymer films can mimic the specific interaction between antibody and antigen, allowing the monomer to trap the template molecule during the polymerization process and to form three-dimensional imprinted sites for the specific recognition of the target molecule [292]. Due to these properties, they are widely designed into various molecular-specific templates while offering higher sensitivity. In the cases of urea detection, poly (methyl methacrylate) PMMA and urea were used as functional polymers and molecular templates, respectively, to fabricate the MIPs on the ion-sensitive BioFETs. The response voltage level is higher than that of conventional Si_3_N_4_-based BioFETs, which implies a great improvement in the sensitivity and selectivity for urea detection [275]. MIPs work in the detection of unamplified samples as well. The direct binding of the MIP to the GFETs resulted in the generation of additional electronic labels, allowing the device to observe characteristic bumps in the noise spectrum only in the presence of the target molecule. This property enables the MIP-based GFETs to have a serotonin LOD two orders of magnitude higher than the existing reports [132].

MIP-based BioFET performance can be analyzed and optimized through Langmuir adsorption isotherm equation. This formula has been used to evaluate parameters such as the binding affinity between small biomolecules and MIP cavities, and even to show the adsorption mechanism of small biomolecules on specific MIP templates [286], which might benefit the pathological studies of PD metabolism. Glucose detection based on MIP-BioFETs has been fully investigated before. MIP for glucose formed by a glucose template (GluMIP), phenylboronic acids (PBA), and glucose molecules was developed for the detection of low concentrations of glucose in an enzyme-free manner in tears [72]. As diol compounds, the glucose molecules used as templates can be removed under acidic pH conditions upon binding to PBA. Subsequently, BioFETs can detect the density changes of the negative charge generated by the PBA-diol compound [75]. The detected output voltage changes at different sugar concentrations can then be put into the Langmuir adsorption isotherm equation to calculate the LOD. Notably, MIP-based BioFETs can detect glucose about 15–20 times more sensitively than other types of sugars [286].

Based on previous studies and derivations [75], the Langmuir isotherm equation can be described as [286]

(2) $$A=\frac{N[c]}{1+K_{a}[c]}.$$

*A* is the signal observed at equilibrium for the MIP-bound template, while [*c*] refers to the free concentration of the template at equilibrium, *N* is the number of active centers available per unit volume of MIP, and *K_a_* is the binding constant. It can be easily seen that the potential difference at the interface of the electrolyte solution varies with the ion concentration, and in this experiment *K_a_* is the binding constant for glucose and PBA in the MIP solution, and the value of *K_a_* for MIP is an important parameter in controlling the LOD and imprinting factor (IF) [75].

(3) $$K_{a}=\frac{{\left[PBA-Sugar\right]}_{charged}}{{\left[PBA\right]}_{noncharged}[Sugar]}$$

where [*PBA-Sugar*]*_charged_* indicates the concentration of charged PBA-sugar conjugate [72]. Based on this equation, the *K_a_* of *PBA* with glucose was found to be several hundred times higher than values obtained through non-electrochemical detection methods [72]. This enhanced affinity is attributed to the role of MIPs, which function as hydrophilic interfaces with immobilized receptors. By positioning the target molecule closer to the surface, MIPs mitigate the ionic shielding effect, thereby reducing screening and facilitating more accurate measurements [62].

In addition to MIP, the combination of other polymer membranes and BioFETs frees it from the reliance on enzyme-specific detection and transcends ionic shielding length limitations, allowing for continued research as a non-invasive real-time monitoring method in PD systems. Bioreceptors can be directly blended with poly (styrene-co-methacrylic acid) (PSMA) to make a sensing membrane and then be spun-coated onto the extended gate of BioFETs. This method can reduce the ionic shielding length within 0.2 nm [293]. However, when it comes into direct contact with the human body, the design of such chemically synthesized interfacial materials should consider biocompatibility [75]. Meanwhile, several factors of MIPs or other polymer membranes still need to be considered when designing, including the cumbersome polymerization process, complete removal of the template, and deeply embedded template molecules leading to high background signals and narrower detection range, among other issues [292].

In addition to the optimization of immobilization, enhancing the sensitivity can be achieved through reducing Debye screening effect and noise signals caused by non-specific adsorption of interfering species in the solution of biological samples [75]. Most of the current protocols focus on shortening the distance between the BioFET surface and detected biomolecule analytes by either diluting the concentration of the analyte or using a small molecule as a bioreceptor [62,75,294]. However, dilution of the solution may affect the biomolecular conformation, stability or reduced affinity for capture-target molecule interactions [71,98]. More focus can be put on the development of a new interface material structures to realize the efficient signal conduction [45].

The solution to Debye screening can be translated into the filtration of non-specific electrical signals [102]. Theoretically, coating with hydrophilic polymers can inhibit non-specific adsorption of large interfering species such as gate proteins, while preventing small interfering species from approaching the gate surface can be achieved by filter membranes such as polymer nanofilter to improve the sensitivity and specificity of FETs [75]. The polymer nanofilter usually consists of an anchor layer and a filter layer in which the components we added can trap the interference substances. The anchor layer is in contact with the surface of the FET electrode, and target can pass through the filter layer to reach the anchor layer producing a detectable potential change. The polymer nanofilter applied effectively prevents the interaction between protein macromolecules and Au electrodes, thereby reducing non-specific electrical signals. This kind of nanofilter-based FET has been used for the potentiometric detection of small biomolecules [295]. It can further design to be an aptamer-based nanofilter interface, where the filter layer consists of DNA, RNA, or peptide aptamer, which can be used to capture dopamine (DA) molecules. The targets can reach the anchor layer to contact the electrode surface to generate an electrical signal [102]. Devices coated with nanofilter exhibit satisfactory filtration effect and suppress non-specific signals.

These methods enable BioFETs to conduct assays in solutions with high ionic strengths. However, for clinical applications, several factors must be carefully considered to optimize performance. These include the thickness of the polymer layer and the density of immobilized aptamer molecules, which significantly impact assay sensitivity. Additionally, controlling the initial potential of the Au-gated electrodes is crucial to prevent non-specific adsorption of interfering species that could oxidize at the electrodes, among other considerations. To obtain a uniform distribution of the electric field, the materials thickness should be controlled or narrowed down [296], since their oxide thickness, length, orientation and radius play important roles in improving BioFETs sensitivity [297]. In a previous study, Au nanoporous membranes with different sizes, thicknesses, and highly ordered and homogeneous structures were combined to the EGFETs for the hybridization tests for 16S rRNA of Staphylococcus aureus, respectively. The results reveal that the thicker Au membrane layer with smaller diameters promotes a larger hybridization signal [269].

These membranes are then developed to the nanoporous structure since the electrostatic shielding effect is weaker near concave surfaces and stronger near convex surfaces [298]. That is, molecular charges bound to convex portal surfaces (e.g., nanopillars) are more easily screened by counterions in the sample solution and will not induce any electrical signals in FET biosensors [75]. On the other hand, porous membranes (concave surfaces) that allow filtration of biomolecules are more of potential [75]. Bioreceptors embedded in the nanopores of EGFET were verified to enable higher molecular throughput and signal-to-noise ratio [299]. This kind of novel nanosensor combines the advantages of nanopore platforms and BioFETs through the use of nanopipette-based polypyridinium ion FETs [163], which allow real-time adjustment of the nanopore dimensions to fit the size of the target molecule, and even efficient control of molecular transport at the single-molecule level by controlling the gate voltage.

Conventional transducing materials have also been developed to various structures and scientists have been attempting to combine BioFETs with these new nanostructures to enhance the sensitivity of detection because of their larger surface area ratio [62]. For instance, in addition to the SiNWs, SEM analysis indicated that silicon nanoribbon has a width of 120 nm and a height of 25 nm which is larger than the functional surface area of conventional nanowires [296]. SiNW BioFETs can detect protein adsorption and desorption events at the single-molecule level while multiple Si-nanochannels can improve device performance by reducing biochemical noise and minimizing discrete dopant fluctuations [300]. A multiplexed SiNW tunnel FET can independently detect several distinct biomolecules with almost equivalent sensing current levels, regardless of the gold layer position, and have higher sensitivity than traditional BioFETs [301]. Even 120 SiNWs were fixed on the BioFETs for the parallel detection of ctDNA. Due to the high dimensional coherence of 120 SiNWs, they enable signal superposition compared to individual SiNWs, resulting in stronger response signals [302]. Vertically stacked nanosheet gate-all-around BioFETs were developed as well, which showed superior sensitivity compared with nanowire BioFETs or vertically stacked nanowire BioFETs, due to its multi-channel structure [303].

To sum up, methods to enhance the sensitivity and performance of BioFETs-based detection encompass a variety of strategies. These include optimizing probe immobilization techniques, such as enzyme encapsulation, attachment to gold nanoparticles or magnetic beads, and employing MIPs, which offer controlled recognition capabilities and improved detection limits for a wide range of substances, including small molecules. Additional approaches focus on mitigating the Debye screening effect using nanofiltration layers, polymer protective coatings, or passivation layers. Lastly, advancements in synthesis techniques and stringent quality control of transducing materials are crucial for ensuring consistent and high-performance FET-based sensors as well.

#### 4.2.2. Stability and Durability Improvement

The long-term stability and durability of BioFETs are crucial for obtaining accurate and reliable detection results, given that PD is a long-cycle treatment modality. However, stability testing of biosensors in is usually performed prior to or separately from the target receptor-bound biosensing, and the neglect of temporal effects and signal drift, may mask actual biomarker detection and adversely affect device performance [59]. When BioFETs are immersed in physiological environmental solutions for prolonged periods of time, the surface of the SiO_2_ insulating layer may be hydrolyzed by cations, and reactive magazines such as airborne water and oxygen are more likely to be absorbed at the interface, affecting charge injection and transfer, thus affecting the final performance of the device [304]. One way to improve stability and reusability is to separate the sensing area from the electrode area, allowing connections to be made between the two parts, such as extended-gate BioFETs did [57]. Since the sensing area functionalized with bioreceptors is the only part of the device exposed to the environment, this configuration maintains excellent stability and reproducibility. Studies also explored integrating extended-gate BioFETs with electrical sensors to establish redundant validation systems, enhancing reliability in complex environments. The IFN-γ aptamer was immobilized on the extended gate of the BioFETs, which simultaneously serves as the sensing region of the BioFETs and the working electrode for electrochemical detection in the electrolyte solution. Upon encountering the target IFN-γ, its specific binding to the electrode induces changes in the redox peak current of ruthenium hexamine chloride ([Ru(NH_3_)_6_]CI_3_), triggering signal variations in both detection systems. The dual-mode sensor demonstrated higher sensitivity and a broader detection range, effectively mitigating BioFETs response saturation when exposed to high-concentration samples [305].

Apart from the configuration, there have been many cases showing that the precise control of nanoscale thickness through chemical deposition technology helps to stabilize the channel materials. Atomic layer deposition (ALD) is a novel technique for the evaporation of chemical substances, which has been widely utilized in BioFETs design. In current research, ALD was used to control the number of cycles to optimize the thickness and morphology of the generated MoS_2_-SWCNT film for the miRNA detection. This adjustment benefits the charge transfer between SWCNT and MoS_2_ and improves the on/off ratio and mobility of BioFETs. Raman spectra and thermogravimetric analysis proved that ALD enables the growth of MoS_2_ on SWCNT with almost no change and loss of the intrinsic properties of SWCNT, and even helps to remove the polymer PCz that wraps the CNT [306].

In addition to deposition technologies, device surface passivation is more common for enhancing stability and sensor lifetime. For example, even for continuous monitoring under conditions with numerous interferences such as in real biological fluids, BioFETs coated with rGO layer has been proved to be useful [307]. Deposition of an ultrathin layer of Al_2_O_3_ on the SiNW-BioFETs also enabled the device to remain stable in physiological solution for at least four months [308]. Combing the forementioned ALD, another research passivated the entire device with an insulating high-κ dielectric HfO_2_ which greatly improved the sensitivity and long-term stability of BioFETs. In this system, ALD allows thickness control on the nanometer scale and provides excellent uniformity and very low defect density [309]. However, the surface passivation strategy for BioFETs is not without side effects. A current study tried to passivate the metal source and drain of CNT-BioFETs with a patterned epoxy layer (SU-8) and then passivated the entire chip with a thin dielectric. They hoped that the charge-blocking effect created by this double passivation strategy would mitigate the device’s leakage currents and greatly improve the long-term stability of the device, only to find that as the device exhibited more stable detection performance, it usually came with a thicker layer of HfO_2_ (30 nm) and the cost of reduced device sensitivity [59]. Table 5 was proposed to summarize the different solutions mentioned above.

### 4.3. Strategies for Enhancing Clinical Usability

Enhancing the clinical usability of BioFETs is not merely about convenience; it is a critical factor in ensuring their seamless integration into existing PD monitoring workflows and maximizing their impact on patient care. This integration allows for the continuous monitoring of biomarkers and enables adjustments to the frequency and composition of dialysate exchanges based on real-time data, thereby optimizing fluid balance. This is particularly crucial for PD patients, as insufficient fluid removal can result in hypertension and symptoms such as subcutaneous and pulmonary edema, while excessive removal can lead to hypotension and diminished residual renal function [264]. The removal of excess solutes during PD is not selective and unintentional removal of useful substances may occur [10], such as the loss of vitamin D-binding proteins leading to symptoms of malnutrition [310]. Moreover, due to the diversity of factors such as age or diabetes status of different patients, personalized approaches should be explored to address the heterogeneity among different populations and meet the clinical requirements [8].

BioFETs could be miniaturized to provide wearable PoC devices, providing patients with immediate feedback. This is an effective way for managing their health and performing PD-related behavior [311]. These PoC systems demonstrated improvements in health management, as well as albumin and hemoglobin levels [312]. Notably, BioFETs can detect biological targets across a range of human biofluids [70], including not only PDE but also saliva, sweat and tears, making them less invasive compared to traditional plasma or serum tests [228]. No sample preparation are required during BioFETs-based monitoring which is also a unique attribute that adds to its commercial appeal [313]. To develop BioFETs-based devices for PD patients, several aspects must be considered: the multiplexed detection capability, data processing, and the requirements for PoC applications, including wearable design, biocompatibility during use (to minimize adverse immune responses), and device reusability.

#### 4.3.1. Multiplexed Detection Capability

**Figure 12:**
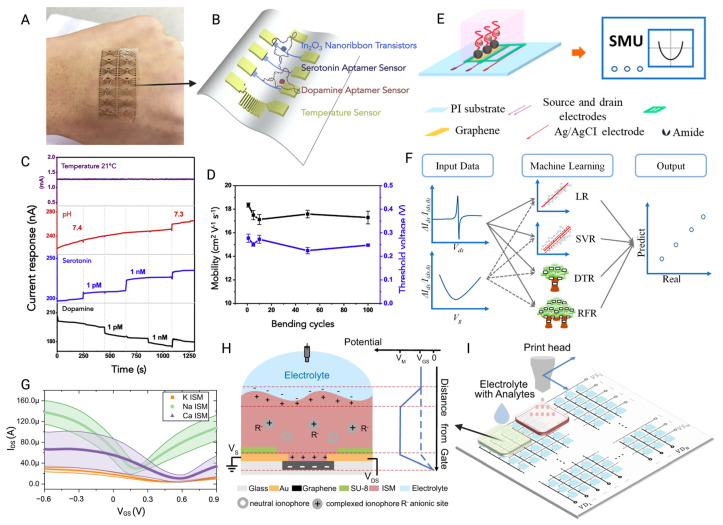
(**A**) The photograph of a flexible sensor array stretched to fit human skin. (**B**) Schematic of an In_2_O_3_ nanoribbon FET for the simultaneous detection of serotonin and dopamine. (**C**) Sensor array simultaneously senses temperature, pH, serotonin, and dopamine changes in artificial cerebrospinal fluid. The two devices were exposed to 1 pM, 1 nM serotonin, 1 pM, and 1 nM dopamine. Both devices responded to the pH change, but only the FET functionalized with the respective target responded to the corresponding target (working under a V_ds_ of 0.2 V and a V_gs_ of 0.25 V). (**D**) Mobility and threshold voltage change curves of the FET sensor in the relaxed state after different bending cycles [148]. (**E**) Schematic of FET-based calibration-free detection of Ca^2+^. The electrical properties of the aptamer-functionalized GFET were measured using a source measurement unit (SMU). (**F**) The measured characteristic curves were analyzed using four machine learning-based algorithms to directly predict the analyte concentration [317]. (**G**) *I_ds_*-*V_gs_* curve of sensing chip measured in electrolyte solution containing 100 μM K^+^, 30 mM Na^+^, and 1 mM Ca^2+^ ions. The solid line is the average value, and the shaded area represents the standard error. (**H**) Single sensing unit with specific ISM. The right curve shows the relationship between the electrostatic potential and the distance from the FET graphene surface. ISM: ion sensitive membrane. (**I**) Schematic diagram of a FET chip composed of N × N sensing units. Different ISMs are printed into different areas for multiplexed detection of different ions [318] (images used under Creative Commons licenses).

The composition of real clinical samples is so complex that the detection of a single analyte is far from sufficient for PD early diagnosis. Developing an efficient method for the simultaneous detection of multiple markers is particularly crucial, especially when these potential biomarkers must be analyzed collectively to assess PD patients’ condition [314]. Integrated biosensing is thought to have the highest accuracy and sensitivity [315], while it can directly convert interactions between biological target analytes and receptors into charge signals without any other labeling process [45]. A unique feature of BioFETs is their miniaturization ability to integrate semiconductor circuits or be in conjunction with microfluidic chips to measure multiple samples at the same time (Figure 12) [75,316]. Constructing a BioFETs array with multiple sensing windows, where each module independently detects a specific biomarker without cross-interference, has been demonstrated to be a feasible approach while maintaining high sensitivity and precision [314]. To create multiple sensing windows, polymer layers such as PMMA were frequently used to spin-coat the array to passivate the source/drain electrodes, leaving a sensing area in the channel using electron beam lithography. Different sensing windows were then functionalized separately with specific antibodies to enable independent biomarker detection [314].

Compared to sensor array, integrating microfluidics and immunoassays into lab-on-a-chip [319] devices is more effective and standardized. Microfluidic technology is frequently used to integrate multiple BioFETs into one system and guide samples to the channel positions of different BioFETs to achieve specific binding and differentiation of targets [320]. It helps to detect biomarkers in shorter response time and with lower reagent consumption [45], which contributes to the prototype development of a miniaturized rapid PoC platform for PD routine monitoring. The fabrication of microfluidic systems typically involves coating a Si wafer with photoresist, patterning the microfluidic structures using a photomask, sealing them with PDMS, and bonding the system to BioFETs via O_2_ plasma treatment [268]. Alternatively, many studies employed 3D printing to directly fabricate polycarbonate as the top and bottom layers of the chip, using a polyimide film as the channel and sensing layer. The source, drain, and gate electrodes of the BioFETs are then patterned on the PI film through photolithography and thermal deposition [321]. Through this technology, a study integrated two different antibiotics-functionalized BioFETs into a dual-channel microfluidic chip to distinguish between GPB and GNB. The portable BioFETs exhibited unprecedented high sensitivity and real-time target specificity with a LOD of 100 CFU/mL, which would facilitate physicians to quickly make a judgement on the type of bacterial infections during PD, reducing the likelihood of other complications as well as the overdose of antibiotic usage [268]. Another study developed a dual-mode diagnostic platform based on a microfluidic chip using 3D printing, integrating electrochemiluminescence (ECL) sensors and BioFETs within the microfluidic device. The microfluidic channels guide the test samples through the electro-optical sensor units, and similar to the previous setup, the gate electrode of the FET is also used as the working electrode for the ECL sensor. Due to the different signal transduction mechanisms, the combined ECL and FET sensors do not interfere with each other in practical tests. To further reduce uncertainties in complex testing environments, the dual-mode output is refined using machine learning (ML) algorithms to denoise the incoming signals and construct a three-dimensional hyperplane that facilitates multi-class diagnosis. Finally, the platform demonstrated diagnostic accuracy of 99% for 501 clinical samples of tuberculosis (TB), human rhinovirus (HRV), and Group B Streptococcus (GBS) from serum and nasopharyngeal/rectal swabs, whereas existing PoC technologies achieved diagnostic accuracy of 77–93% at 100% statistical power [321].

To improve the reliability of sensor arrays and precisely differentiate signals from multiple analytes, various algorithms were implemented to optimize both the detection and analysis processes, including those refine signal clarity by processing interference [322] and compensate for device variations caused by imperfections in material synthesis and inconsistencies in device fabrication [318]. While combined systems provide two categories of clinical data (positive and negative results), in practice, there are ambiguous samples that cannot be identified by the combined system, thus transforming the problem into a multi-class classification task [321]. For example, in one previous study, a dual-channel GFET was designed to simultaneously detect dopamine (DA) and glucose. The sensor utilized 11-mercapto-1-undecanoic acid-gold nanoclusters (MUA-AuNCs) and PBA to enhance the selectivity and sensitivity of the FET for DA and glucose detection. To differentiate the signals from the two analytes, a unique data fusion algorithm based on a least squares method was employed. This approach utilized precise DA readings from one channel to correct for interference and calculate the accurate glucose concentration in the mixed solution, thereby ensuring high detection accuracy [322].

Building on the previous example of data fusion algorithms, another study employed a different approach to enhance the performance of GFET-based sensors. Three distinct ion-selective membranes (ISMs) were integrated onto the GFET to selectively recognize potassium, sodium, and calcium ions in urine and sweat. The GFET was composed of over 200 integrated sensing units (16 × 16) and divided into three separate regions, which made it challenging to accurately quantify the concentration of three ions. To address this issue, the team utilized a source measure unit (SMU) to assess the electrical performance of the fabricated BioFETs and confirmed their typical output and transfer curves. Subsequently, various algorithms were employed to establish regression models, including linear regression (LR), support vector regression (SVR), decision tree regression (DTR), and random forest regression [323], with the goal of analyzing their respective feature curves. By evaluating metrics such as mean square error [115], root mean square error (RMSE), mean absolute error, and R^2^, it was found that the RFR model exhibited the best performance in estimating Ca^2+^ concentration without the need for calibration, with the predicted concentrations closely aligning with the actual concentrations (y = x). The integration of these algorithms not only provided a calibration method to reduce device-to-device variability, but also enhanced the accuracy of ion classification, further improving the performance of the GFET-based sensor system [317]. Subsequent studies employed RFR to extract additional parameters reflecting the changes in graphene films following deposition. Beyond the Dirac point [324], other features derived from the I-V curve, such as the maximum and minimum transconductance (for both electrons and holes), were analyzed to study the physical property variations of graphene. Compared to other algorithms that are also compatible with multiplexed sensor chip data analysis, such as deep neural networks (DNN) and support vector machines (SVM), RFR, owing to its tree-based structure, is more interpretable and requires less data. This makes RFR particularly suitable for analyzing data sets from sensor chips with fewer variables or simpler data structures [318].

#### 4.3.2. Development of PoC Devices

**Figure 13:**
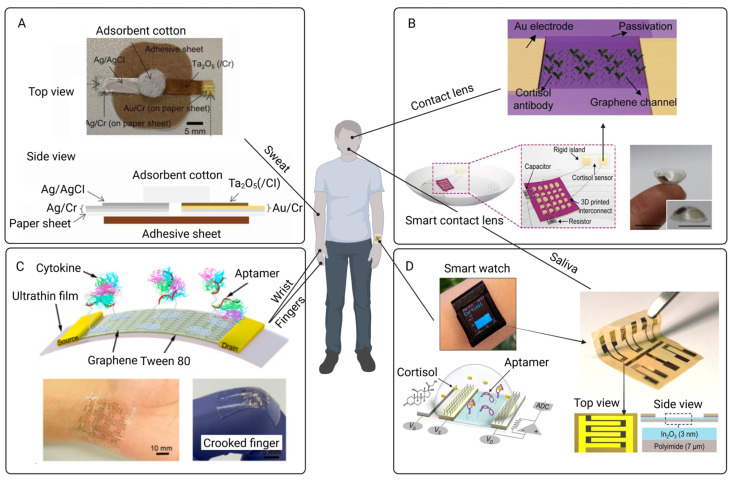
Wearable BioFETs for the detection of biomarkers in different human fluids. (**A**) Schematic of a wearable BioFET patch for assessment of Na^+^ concentration in sweat, where a plasticizer-free ISM based on fluoropolysiloxane (FPS) improves biocompatibility [327]. (**B**) Schematic of smart contact lens modified with antibody for the cortisol detection [328]. (**C**) Schematic of an ultra-flexible GFET for the cytokine detection in biofluids. The flexibility and stretchability of GFET allows it to attach onto the human wrist and crooked fingers [279]. (**D**) Top: schematic of a smart watch functionalized by aptamer-based FET for the cortisol detection in saliva and sweat samples. Bottom: Schematic of FET structure. Right: top view and side view of In_2_O_3_-based FET with a flexible polyimide substrate. ADC, analog-digital converter [129] (images used and edited under Creative Common licenses).

With further research into PD, additional physiological fluids—such as saliva—emerged as potential alternatives for monitoring metabolic by-products associated with renal failure. Moreover, tears and sweat could also be considered physiological solutions for assessing creatinine or glucose levels during PD [207]. BioFETs hold significant potential for developing wearable devices tailored to PD patient monitoring through these various fluids, enabling personalized adjustments to treatment regimens. They have been incorporated with different flexible materials and designs to ensure wearability (Figure 13).

For instance, the extended gate of the BioFETs was designed as a microneedle to penetrate the skin and measure Na^+^ in the skin interstitial fluid when worn [325]. In their study, researchers first created solid polystyrene microneedles using PDMS molds. These microneedles then served as the base for depositing gold electrodes, while silver was applied to another microneedle electrode to function as a reference electrode. The stretchable design was achieved by patterning AgNWs into elastomeric SIS substrate. Thanks to the interconnected network structure and high conductivity of AgNW, the electrodes continue to perform reliably even when bent, twisted, or stretched. Mechanical tests using SIS films with a thickness gradient loaded with these rigid microneedles demonstrated that, even when the patch is stretched to 100% strain, the central region remains intact and flexible. During the detection process, the BioFETs convert the concentration input into an electrical signal, which can be sent directly to doctors and clinics via Internet-of-Things-enabled smartphones or computers. ISFET was integrated with microfluidics to make a wearable sweat analyzer to track the Na^+^ and K^+^ concentrations changes in sweat in real time. An SU-8 passivation layer was deposited on the top of the fabricated ISFET and patterned to create an integrated passive microfluidic interface. This setup leverages capillary action to direct biological fluids into an array of functionalized ISFETs for detection and transport to the outlet area [326]. Furthermore, nanoFETs were incorporated into injectable electronics or direct implantation into specific tissues for vivo monitoring or intracellular measurements, such as the development of SiNW-BioFETs arrays embedded in engineered tissue patches [294].

The biocompatibility of these BioFETs should be highlighted to prevent adverse reactions. For instance, while higher sodium-to-creatinine ratios are often associated with the progression of end-stage renal failure [329], ISMs are frequently combined with wearable BioFETs to detect Na^+^ in sweat, in which ISMs are drop-cast on top of the sensing dielectric. The ionophores in the ISM interacted with the target ions with high selectivity to reduce the non-specific adhesion [330]. However, this system usually needs a reference electrode [331], so CNT was then incorporated with ISFETs. Ag/AgCl ink and polyvinyl butyral membrane were used to fabricate an integrated reference electrode, and the CNT surface was functionalized with a polyvinyl chloride (PVC)-based ISM. The BioFETs can detect sodium ions in the range of 0.1 to 100 mM with high sensitivity. This structure solved the problem of large size of the traditional ISFETs reference electrode, and it can provide a stable potential comparable to that of the commercial reference electrode [332]. However, PVC-based ISMs usually include a plasticizer, and if ISM-coated sensors are used to monitor health parameters in daily life, prolonged immersion of the plasticizer in solution may lead to cytotoxicity and shorter sensor lifetime [333]. New strategies have been developed using Ta_2_O_5_ oxide membranes and fluoropolycrystalline silicon (FPS) to fabricate the plasticizer-free ISMs, and the device exhibited higher biocompatibility as well as retained sufficient sensitivity and selectivity to Na^+^ [327]. The combination of biocompatible and flexible coating materials such as hydrogels or liquid metals with these systems has been proved to minimize immune reactions and potential tissue damage as well [334]. Additionally, these coatings contribute to enhance the sensitivity of BioFETs as mentioned above. Spin-coated hydrogels synthesized by free radical polymerization were deposited on SiNW BioFETs serving as dielectric and functional layers. Compared to conventional SiNW BioFETs, the ones modified with hydrogel coatings have higher biocompatibility and time response [335].

Building on the advancements in biocompatibility and flexible designs, reusability is another essential aspect of PoC devices that ensure long-term functionality and cost-efficiency for patients and clinicians. The primary requirement for reusable sensors is the regenerability of probe–analyte interactions. Methods such as acid base rinsing, heating, and voltage biasing are commonly employed to achieve in situ dissociation of probe–analyte complexes [336]. For instance, one approach leverages the reversible interaction between PBA and glucose. In acidic conditions, the bonds between glucose and PBA are disrupted, allowing glucose molecules to dissociate from boronate ester complexes and regenerating the device surface for subsequent cycle [130].

Several other innovative strategies further enhance the reusability of FET-based biosensors. In the urea detection, back-gated BioFETs were placed within a sealed configuration to measure the urea. Urea molecules are enzymatically dissociated into ammonia gas, enabling reversible adsorption-desorption cycles that facilitate multiple uses without significant performance loss [274]. In another study, they immobilized the cortisol antibody onto the magnetic beads. Following analyte detection, the magnetic nanoparticles can be removed using an external magnetic field, effectively resetting the sensor surface while preserving the functional integrity of the device [136]. Lastly, a novel approach has been demonstrated with highly durable and reusable drug molecule-based OECTs (DM-OECTs). In this system, protonated gefitinib is electrostatically adsorbed onto the PEDOT:PSS layer. The introduction of competitive gefitinib-EGFR binding induces additional surface gating and de-gating of the active layer, creating a dynamic and refreshable sensing interface [336]. These strategies exemplify the potential of innovative reusability solutions to extend the operational lifespan and functionality of PoC devices, furthering their applicability in real-world diagnostics.

## 5. Concluding Remarks

BioFETs offer promising advancements for the field of PD by enabling precise and real-time monitoring of critical biomarkers, which is essential for personalized patient care. By addressing the sensitivity and selectivity challenges posed by the complex physiological environment, BioFETs can be optimized to deliver accurate biomarkers detection such as urea, creatinine, inflammatory markers, and bacterial infections, enhancing their clinical relevance for daily monitoring. However, the heterogeneous composition of PDE and the high-ionic environment pose considerable challenges to the performance of BioFETs, underscoring the need for stable and specific transducing materials that can maintain functionality over extended clinical use.

The scalability of BioFET fabrication and integration into existing PD frameworks are equally vital for transitioning from laboratory prototypes to clinically viable devices. Advances in nanomaterials, such as MoS_2_ and carbon-based technologies, alongside cost-effective manufacturing processes, including CMOS and CVD compatibility, will be instrumental in making BioFETs accessible at a commercial level. Additionally, the development of wearable, PoC BioFETs offers a novel approach to continuous and minimally invasive monitoring across various biofluids, facilitating frequent and accessible assessments for PD patients.

Future work should prioritize embedding BioFETs into closed-loop PD systems, where real-time biomarker data from BioFETs automatically adjust dialysate composition via feedback-controlled pumps. Such integration would minimize human error and optimize solute clearance. The long-term stability, biocompatibility, and reusability of BioFETs under variable physiological conditions, as well as the integration of multiplexing capabilities to allow concurrent monitoring of multiple biomarkers should be further studies as well. With these improvements, BioFETs could become a cornerstone of patient-centered care in PD, delivering actionable insights and facilitating personalized treatment strategies that ultimately enhance patient outcomes and quality of life. Despite the challenges, the forward momentum in research suggests a transformative future for PD management.

## Figures and Tables

**Table 1:** Comparison of various probe types in FETs for target detection.

Binding Principle	Biosensing Elements	Target	LOD
Enzyme-based	GOD	glucose	200 nM [84]
	GOD/Zno/Cuo	glucose	30 nM [116]
	GOD/MOFs	glucose	0.51 μM [117]
	Urease	urea	1 μM [118]
	SOX	sarcosine	105 zM [119]
	papain	Cys C	0.05 ag/uL [30]
DNA-based	ssDNA	E. coli DNA	1 fM [94]
	SNA	virus RNA	0.13 copies/uL [120]
	DNA	miRNA	100 aM [99]
	PNA	miRNA	0.1 aM [99]
Immuno-based	antibody	TNF-α	1 pg/mL [121]
	antibody	serotonin	0.1 fM [122]
	antibody/AuNPs	IL-6	2.2 fM[123]
	antibody	Cys C	0.25 ag/mL [124]
	antibody/casein	E. coli	1 CFU/mL [125]
	antibody	CRP	0.06 μg/mL [35]
	antibody fragment	CRP	0.73 μg/mL [126]
Aptamer-based	aptamer	glucose	0.5 fM [127]
	aptamer	IL-6	618 fM [128]
	aptamer-SH	cortisol	1 pM [129]
Other binding events-based	PBA	glucose	0.15 μM [130]
	GO/Ag	glucose	1 μM [131]
	MIP	serotonin	0.05 fM [132]
	MIP	TNF-α	0.55 pg/mL [133]

GOD, glucose oxidase; LOD: limit of detection; MOF, metal-organic frameworks; SOX, sarcosine enzyme; Cys C, cystatin C; ssDNA, single-stranded DNA; SNA, spherical nucleic acids; PNA, peptide nucleic acid; CRP, C-reactive protein; PBA, phenylboronic acid; GO, graphene oxide; and MIP, molecularly imprinted polymer.

**Table 2:** Examples of transducing materials and modifications in enhancing FET performance.

Type	Transducing Material (Modification)	Target	Remark	Sensitivity
Bulk	Si (Ge)	/	Si_0.7_Ge_0.3_	Electric performance: 2 × higher sensitivity vs. pure Si [144]
	Bulk graphene (AuNPs)	/	Higher defect density of bulk graphene	ON/OFF current ratio: 4 × higher [146]
MOS	In_2_O_3_ (Al_2_O_3_)	/	Surface passivation	low operation voltage (0.05 V) [152]
	In_2_O_3_ (Al_2_O_3_/SU-8)	/	Passivation and protection layer	low operation voltage (0.005 V) [153]
1D	Si NWs	Exosome	45 nm width poly-Si nanowires	2159 particles/mL [50]
	Si NWs	Virus DNA	10 Si nanowires	1.93 fM [159]
	Si NWs	CRP	Antibody fragment as probe	0.73 μg/mL [126]
	SWCNT	CRP	Suitable for minute quantities of analytes	0.06 μg/mL [35]
	Si nanosheet	CRP	Vertically stacked channels	100 pg/mL [175]
	SCNT (liquid silver)	DNA	Suspended material mitigates the impact of the substrate	10 aM [176]
	MWCNT (PEI/ZrO_2_)	/	ZrO_2_: decrease current leakage; PEI: increase CNT conductivity [69]	/
2D	Graphene (plasma)	DNA	Plasma treatment removes residues and enhances the hydrophilicity	10 aM (an order of magnitude higher vs. unmodified) [177]
	Graphene (Monolayer)	DNA	CVD-grown graphene	15 fM [166]
	Graphene (defect-engineered)	Glucose	More binding sites introduced with lower energy barriers	Glucose sensitivity: 0.16 mV/mM higher vs. unmodified [170]
	Graphene (polymerized)	Glucose	Reversible reaction between glucose and polymer	1.9 μM [178]
	rGO	miRNA	Easier to modify	1 fM [179]
	WS_2_ (monolayer)	/	MOCVD minimizes the contamination during film transfer	33 cm^2^ V^−1^ s^−1^ of carrier mobility (1.5 times higher than the best reported) [180]
	MoS_2_ (nanoporous)	Cortisol	More binding sites and edges	1 ag/mL [172]
	Single-layer MoS_2_ (Pt)	Cortisol	Stronger charge transfer effect [181]	/
	MXene	CD9/Exosome	Tunable properties and the numerous functional groups on MXene	CD9: 10.64 pM Exosome: 6.41 × 10^2^ exosomes/mL [101]
	Black phosphorus	Cortisol	Adjustable band gap	1 aM [136]

Si NWs, silicon nanowires; CRP, C-reactive protein; SWCNT, single-walled carbon nanotube; SCNT, suspended carbon nanotube; MWCNT, multi-walled carbon nanotube; PEI, polyethylene imine; CVD, chemical vapor deposition; rGO, reduced graphene oxide; and MOCVD, metal–organic chemical vapor deposition.

**Table 3:** Examples of transducing materials and modifications in enhancing the performance of BioFETs.

Biofluids	Biomarker	Disease/Application	Remarks	Ref.
PDE	Glucose	Ultrafiltration failure/PF	Long-term effects	[243]
PDE/plasma/serum	Urea	Urea removal/renal function	<20 mM during PD	[240]
PDE/serum	Creatinine	Creatinine clearance/renal function	/	[244]
PDE	IL-6	Acute inflammation/PD peritonitis/solute transport rate/EPS	Sharply increase during inflammation	[245]
PDE	CA125	Overhydration/EPS	Peritoneal mesothelial cell count	[246,247]
PDE/serum	HA	PF	Characteristic of PF and wound healing in the peritoneum	[26]
PDE	Water channel Aquaporin 1	Ultrafiltration failure	Increase	[237]
PDE	MMP-2/VEGF	Inflammation/PF/solute transport rate	/	[19]
PDE/urine	Na^+^	Cardiovascular events	Low sodium clearance rate	[241]
PDE/tears/saliva	TNF-α	Inflammation/membrane damage	/	[242]
PDE	Extracellular vesicles	Indicate peritoneal membrane function	Proteome difference	[248]
PDE	lipopolysaccharide	GNB infection	/	[249]
PDE/urine	Decoy receptor 2	PF	/	[17]
PDE/serum	CRP	Inflammation	Sharply increase	[250]
PDE	DNA and miRNA (bacterial and mitochondrial)	Local and systemic inflammation/solute transport rate	/	[251]
PDE/serum	CysC	Acute kidney injury/residual renal function/cardiovascular diseases	/	[252,253]

PDE, peritoneal dialysis effluent; PF, peritoneal fibrosis; PD, peritoneal dialysis; EPS, encapsulating peritoneal sclerosis; HA, hyaluronan; MMP, metalloproteinase; GNB, Gram-negative bacterial; CRP, C-reactive protein; and CysC, cystatin C.

**Table 4:** Examples of BioFETs used for PD-related monitoring.

Application	Target	Probe	Transducing Material	Used Samples	LOD	Ref.
Key Indicators	Glucose	PBA	Copolymerized hydrogel	Buffer solution (PBS)	5 μM	[205]
	Glucose	CuO	CuO nanowires	Slightly basic medium (pH = 7.4)	1 mM	[271]
	Glucose	Enzyme	PEG/SiNW	Buffer solution (PBS)	10 nM	[77]
	Glucose	Enzyme	Vertical ZnO nanorods	Buffer solution (PBS)	0.05 mM	[272]
	Glucose	Peptide hydrogel	In_2_O_3_	Glucose samples	10 nM	[273]
	Glucose	PBA	MIP	Buffer solution (sodium phosphate)	3 μM	[72]
	Creatinine	Enzyme	MWCNT	Buffer solution (pH = 7.4)	/	[228]
	Creatinine	Enzyme	Silicalite	Buffer solution (KH_2_PO_4_-NaOH)	5 μM	[231]
	Urea/ammonia	CdS/TiO_2_	CdS/TiO_2_	Urine samples	0.85 ppm	[274]
	Urea	Urease	SnO_2_/IGZO	Buffer solution (PBS)	/	[219]
	Urea	MIP	Si_3_N_4_	Urea samples	1.0 × 10^−4^ M	[275]
	Urea	Urease	Si	Buffer solution (PBS)	/	[139]
	Uric acid	Uricase	RuO_2_	Buffer solution (PBS)	0.082 mg/dL	[276]
	Uric acid	MoS_2_	MoS_2_	IPA solvent	60 nM	[277]
Potential Infection Biomarkers	IL-6	Antibody	SWCNT	Buffer solution (PBS)	1.37 pg/mL	[255]
	IL-6	Aptamer	HfO_2_/graphene	Saliva	12 pM	[187]
	IL-6	3’-thiolated aptamers	MXene	/	10 fg/mL	[257]
	CA125	Aptamer	MWCNT	Buffer solution (PBS)	0.5 nU/mL	[261]
	CA125	Antibody	InSe	/	0.01 U/mL	[262]
	Albumin	Antibody	SWCNT	Human serum	0.47 fg/mL	[112]
	TNF-α	Antibody	Si_3_N_4_	Artificial saliva and Buffer solution (PBS)	/	[278]
	TNF-α	Aptamer	Graphene	Artificial tears	1 nM	[266]
	TNF-α and IFN-γ	Aptamer	Graphene	Artificial tears	2.75 pM/2.89 pM	[279]
	CRP	Fab	SiNW	Buffer solution (PBS)	0.6 μg/mL	[250]
	CRP	Antibody	CNT	Buffer solution (PBS)	0.06 μg/mL	[35]
	Cystatin C	Papain/AuNPs	LIG	Urine samples	0.05 ag/μL	[30]
	DNA	DNA probe	MoS_2_/graphene	Buffer solution (PBS)	10 aM	[280]
Germs Infection	*S. aureus* and *S. epidermidis*	Fibronectin	SAMs	Buffer solution (PBS)	9 × 10^5^ CFU/mL	[56]
	*S. aureus*	Polystyrene nanospheres	Au nanoporous structure	Buffer solution (PBS)	1 pM	[269]
	*S. aureus*	Antibody	SWCNT	Buffer solution (PBS)	150 CFU/mL	[109]
	GPB and GNB	Antibiotics	Graphene	Bacterial sample	1–9 CFU/mL	[268]
	*S. aureus*	RCD	SWCNT	Buffer solution (PBS)	1 CFU/mL	[270]

PBA, phenylboronic acid; PEG, polyethylene glycol; MIP, molecularly imprinted polymer; MWCNT, multi-walled carbon nanotube; SWCNT, single-wall carbon nanotube; CRP, C-reactive protein; LIG, laser-induced graphene; SAMs, self-assembled monolayers; GPB, Gram-positive bacterial; GNB, Gram-negative bacterial; and RCD, RNA-cleaving DNAzyme.

**Table 5:** Comparison and solutions to overcome technical barriers.

Principle	Solutions	Advantages	Disadvantages
Loading matrix	Microbeads	replaceable	Partial depletion of enzyme
	AuNPs	Improved stability/catalytic efficacy	High cost for mass-manufacturing
	Nafion/silicalite	Improved stability	Substrate diffusion affected/enzyme activity affected due to pH
Artificial membrane	Polymer membrane (e.g., MIPs, PSMA)	Higher sensitivity/specificity/reduced ion screening effect	Biocompatibility concerns/complex synthesis
Reduction in non-specific signals	Dilution	Easy to manipulate	Affinity of biomolecules/stability/sensitivity might be affected
	Polymer nanofilter	Reduced non-specific signals	Concerns of membrane thickness/size/orientation control/poor reproducibility
Larger reaction area	Porous structure	More binding sites/improved sensitivity	
	Nanowires/nanoribbon. etc.	Fit in multichannel design/higher sensitivity due to larger surface	
Improved stability	Extended-gate design	Improved stability and reusability	Potential signal loss/complex integration
	Deposition technology (e.g., ALD)	Optimized thickness/morphology control/interface quality	Higher cost and complexity/slow deposition speed
	Passivation/protection layer (e.g., rGO, HfO_2_, and AI_2_O_3_)	Improved stability and biofunctionalization/high dielectric constant/mature technology	Non-uniform deposition/dielectric and interface trade-offs/thickness control

AuNPs, gold nanoparticles; MIPs, molecularly imprinted polymers; PSMA, poly (styrene-co-methacrylic acid); ALD, atomic layer deposition; and rGO, reduced graphene oxide.

## Data Availability

No new data were created or analyzed in this study. Data sharing is not applicable to this article.
